# Omega‐3 polyunsaturated fatty acids and its metabolite 12‐HEPE rescue busulfan disrupted spermatogenesis via target to GPR120

**DOI:** 10.1111/cpr.13551

**Published:** 2023-09-24

**Authors:** Jun Jing, Lei Ouyang, Hong Zhang, Kuan Liang, Rujun Ma, Xie Ge, Ting Tang, Shanmeizi Zhao, Tongmin Xue, Jiaming Shen, Jinzhao Ma, Zhou Li, Jing Wu, Yang Yang, Wei Zhao, Lu Zheng, Zhang Qian, Shanshan Sun, Yifeng Ge, Li Chen, Chaojun Li, Bing Yao

**Affiliations:** ^1^ State Key Laboratory of Reproductive Medicine and Offspring Health Nanjing Medical University Nanjing China; ^2^ Department of Reproductive Medicine, Affiliated Jinling Hospital Nanjing Medical University Nanjing China; ^3^ Department of Reproductive Medicine, Affiliated Jinling Hospital, Clinical School of Medical College Nanjing University Nanjing China; ^4^ Department of Reproductive Medicine, Affiliated Jinling Hospital, The First School of Clinical Medicine Southern Medical University Nanjing China; ^5^ School of Life Science Nanjing Normal University Nanjing China; ^6^ Reproductive Medical Center, Clinical Medical College (Northern Jiangsu People's Hospital) Yangzhou University Yangzhou China; ^7^ Core Laboratory, Sir Run Run Hospital Nanjing Medical University Nanjing China; ^8^ Basic Medical Laboratory, Affiliated Jinling Hospital, Clinical School of Medical College Nanjing University Nanjing China

## Abstract

Busulfan is an antineoplastic, which is always accompanied with the abnormal of spermatogonia self‐renewal and differentiation. It has been demonstrated that the omega‐3 polyunsaturated fatty acids (PUFAs) benefits mature spermatozoa. However, whether omega‐3 can protect endogenous spermatogonia and the detailed mechanisms are still unclear. Evaluate of spermatogenesis function (in vivo) were examined by histopathological analysis, immunofluorescence staining, and western blotting. The levels of lipid metabolites in testicular tissue were determined via liquid chromatography. We investigated the effect of lipid metabolites on Sertoli cells provided paracrine factors to regulate spermatogonia proliferation and differentiation using co‐culture system. In our study, we showed that omega‐3 PUFAs significantly improved the process of sperm production and elevated the quantity of both undifferentiated Lin28+ spermatogonia and differentiated c‐kit+ spermatogonia in a mouse model where spermatogenic function was disrupted by busulfan. Mass spectrometry revealed an increase in the levels of several omega‐3 metabolites in the testes of mice fed with omega‐3 PUFAs. The eicosapentaenoic acid metabolite 12‐hydroxyeicosapentaenoic acid (12‐HEPE) up‐regulated bone morphogenic protein 4 (BMP4) expression through GPR120‐ERK1/2 pathway activation in Sertoli cells and restored spermatogonia proliferation and differentiation. Our study provides evidence that omega‐3 PUFAs metabolite 12‐HEPE effectively protects spermatogonia and reveals that GPR120 might be a tractable pharmacological target for fertility in men received chemotherapy or severe spermatogenesis dysfunction.

## Graphical abstract

This study demonstrates that omega‐3 effectively protects spermatogonia from the effects of chemotherapy and oxidation; specifically, it rescues spermatogonia function by enhancing bone morphogenic protein 4 (BMP4) expression in Sertoli cells. We further found that some eicosapentaenoic acid metabolites, including 12‐hydroxyeicosapentaenoic acid (12‐HEPE), were produced at high levels in the testes of mice administered omega‐3 and that 12‐HEPE specifically induced BMP4 expression through the GPR120‐ERK1/2 signalling pathway in Sertoli cells.

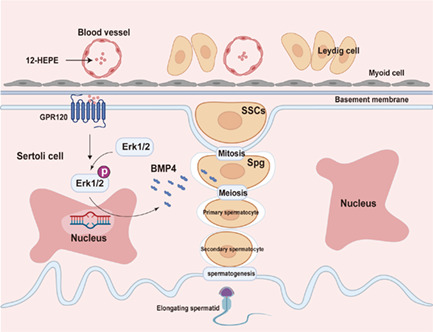

## 1 INTRODUCTION

The male reproductive toxicity of busulfan, which is a crucial drug for treating cancer, is widely known. Busulfan can result in loss of spermatogonia number and function,^1^ and lead to male temporary or permanent sterility.^2^, ^3^ There are no medically endorsed treatments available to enhance spermatogonia function in a non‐invasive and safe manner, so it is critical to identify a good pharmaceutical strategy to minimize negative impact of busulfan.

The regulation of spermatogenesis is controlled by the peculiar microenvironment found in the testes, consisting of Leydig cells, peritubular myoid cells, Sertoli cells, and the cytokines they secrete.^4^ Sertoli cells, acting as the main supporting cells, offer structural assistance, nutritional maintenance, and paracrine substances to control the mitosis of spermatogonia, meiosis of spermatocytes, and the conversion of round spermatids into spermatozoa.^5^ Studies have shown that Sertoli cells release bone morphogenic protein 4 (BMP4), glial cell line‐derived neurotrophic factor (GDNF), neuregulins (NRGs), and Kit ligand (KitL), which regulate spermatogonial stem cell (SSC) self‐renewal and differentiation.^6^, ^7^, ^8^, ^9^ Abnormal secretion of paracrine factors from Sertoli cells may lead to spermatogenesis dysfunction, which may eventually cause male infertility.^10^ However, the mechanism by which paracrine factors in the microenvironment regulate spermatogenesis is still unclear, thus elucidating this mechanism might provide targets for the treatment of spermatogenic dysfunction.

Male reproductive system contains testes and sperm, which possess a unique lipid makeup and are abundant in omega‐3 polyunsaturated fatty acids (PUFAs). These omega‐3 PUFAs may have an impact on reproductive function and fertility.^11^ Many studies have shown that essential omega‐3 PUFAs, mainly docosahexaenoic acid (DHA) and eicosapentaenoic acid (EPA), cannot be produced in most animals, especially humans de novo.^12^ In particular, PUFAs and their byproducts not only function as secondary messengers within the cellular membrane but also demonstrate a diverse array of biological activities.^13^, ^14^ The DHA and EPA compounds must be delivered to the body as therapeutic formulations or be consumed from dietary sources. The delivery systems of omega‐3 PUFAs‐containing three‐dimensional supramolecular lipid assemblies can increase their bioavailability of pharmaceutical applications.^15^ Several clinical studies have suggested that supplementation with omega‐3 improves sperm count and function.^16^, ^17^ Numerous researches have indicated that the level of unsaturation of PUFAs is associated with the fluidity and flexibility of the membrane, as well as the function of receptors and the achievement of successful fertilization in mature spermatozoa.^18^ Studies have shown that deficiency of PUFA elongation or desaturation enzymes in Sertoli cells and spermatogenic cells has been linked to impaired spermatogenesis.^19^, ^20^, ^21^ The concentration of omega‐3 PUFAs in semen is notably reduced in oligoasthenospermia patients compared with in healthy men,^22^ suggesting that omega‐3 PUFAs might have a crucial function in controlling spermatogenesis. Hence, it is necessary to conduct additional research on the local regulatory effect of omega‐3 PUFAs and its metabolites on the microenvironment during spermatogenesis, and whether EPA/DHA themselves or their metabolites have a beneficial effect should be explored.

A mouse model of busulfan‐disrupted spermatogenesis and an in vitro co‐culture system was utilized to explore the possible role of omega‐3 PUFAs in spermatogenesis. The results of our study indicate that omega‐3 mainly has a safeguarding effect on the reproductive system through its metabolites, triggering a signalling cascade of G protein by interacting with G protein‐coupled receptor 120 (GPR120), which may be involved in ERK activation, thereby promoting the paracrine function of Sertoli cells and regulating spermatogonial cell proliferation and differentiation. Our results suggest that omega‐3 metabolites can protect the reproductive system, providing new ideas and targets for clinical treatment.

## 2 MATERIALS AND METHODS

### 2.1 Acquirement of human seminal plasma sample

Between the hours of 8:00 and 11:00 AM, we collected samples of serum and seminal plasma from a total of 50 individuals. Among them were 25 patients diagnosed with non‐obstructive azoospermia (NOA; *n* = 21) or extreme oligospermia (EO; *n* = 4), and the remaining 25 men were healthy individuals serving as controls. These samples were obtained during routine semen analysis at our clinical laboratory, specifically between September 2017 and January 2020. Please refer to Table S1 for further details. The age range of the individuals was between 20 and 43 years. After being subjected to centrifugation at room temperature for 10 min at a force of 1800 times the acceleration due to gravity, the serum samples were subsequently preserved at −70°C. After centrifuging the seminal plasma samples at a force of 12,000 times gravity for a duration of 5 min, they were subsequently stored at a temperature of −70°C. The iFlash Chemiluminescence Immunoassay Analyser (YHLO Biotech, Shenzhen, China) was utilized to measure the levels of inhibin B in the serum. The analysis of sperm quality was conducted by employing a computer‐assisted system called WLJY‐9000 (manufactured by WeiLi Co., Ltd, Beijing, China). Sperm criteria were deemed normal if the concentration of sperm exceeded 15 × 10^6^/mL, the progressive motility showed more than 32%, the total motility was above 40%, and the rate of normal sperm morphology was higher than 4%. NOA was determined when there was an absence of sperm in either the analysis of semen or the biopsy of the testicles, without any abnormalities in the chromosomal karyotype or deletion in the AZF region. Informed consent was obtained before collecting all clinical samples, and the Research Ethics Committee of Jinling Hospital approved the protocol.

### 2.2 Analysis of fatty acids in seminal plasma

The levels of free fatty acids in seminal plasma were examined using gas chromatography coupled with mass spectrometry (GC/MS). In summary, the specimens were defrosted on ice and supplemented with a solution of chloroform and methanol. Following the application of ultrasonication, the liquid portion was gathered. Next, a solution of sulfuric acid‐methanol with a concentration of 1% was introduced, and the mixtures were left to incubate at a temperature of 80°C for a duration of 30 min to facilitate the esterification of free fatty acids. Afterwards, the methylated fatty acids were obtained by extracting them using n‐hexane and then rinsing them with water. GC/MS was utilized to determine the concentrations of methylated fatty acids employing an Agilent 7890A/5975C GC/MS equipped with an Agilent DB‐WAX capillary column (30 m × 0.25 mm ID × 0.25 μm) (Agilent Technologies, CA). The analysis involved quantifying the quantities of 39 free fatty acids with medium and long chains (specifically, C6‐C24). Shanghai Applied Protein Technology conducted this analysis.

### 2.3 Animal models

Male mice of the C57BL/6J strain, aged 5 weeks, were acquired from Beijing Vital River Laboratory Animal Technology Co. They were kept in a controlled environment with a temperature range of 20–26°C and a light/dark cycle of 12 h each. For every experiment, a total of six groups were formed by randomly assigning 6‐week‐old mice (*n* = 6 per group) using the random number technique. All mice used in the experiment were housed in the animal facility located at Jinling Hospital. The Animal Care and Use Committee of Jinling Hospital approved all animal experiments, which were conducted in compliance with institutional guidelines. To establish a mouse model of spermatogenesis disorder, an intraperitoneal injection of busulfan at a dose of 35 mg/kg was given to mice as a single dose, aiming to disrupt the endogenous germ cells. Meanwhile, the control group was treated with intravenous injection of dimethyl sulfoxide. Following the administration of busulfan, the mice received a 12‐week consecutive gavage of omega‐3 (produced by Chengdu Gowell Pharmaceutical Co., Ltd.) at a dosage of 2 g/kg/2 days. To investigate whether the effects of 12‐hydroxyeicosapentaenoic acid (12‐HEPE; cat no. GC40359, Glpbio; 100 μL, 2 μM/2 days) on spermatogenesis in the busulfan disrupted model could be inhibited by the GPR120 antagonist AH7614 (cas no. 6326‐06‐3, Selleck; 100 μg/2 days), the mice were treated with 12‐HEPE (gavage) with or without AH7614 (intraperitoneal injection) for 12 consecutive weeks.

### 2.4 Assessing the counts and motility of sperm in mice

Spermatozoa from a section of the cauda epididymis were discharged into 0.5 mL of human tubal fluid solution with 0.5% bovine serum albumin and maintained for 5 min at a temperature of 37°C. Immediately after collecting the sperm, the standard method was used to determine sperm motility on a haemocytometer under a light microscope. The percentage of moving sperm cells was determined by calculating the number of mobile spermatozoa per unit area. Sperm counts were additionally assessed utilizing a haemocytometer, and the findings were presented as million mL^−1^ of suspension.

### 2.5 Histopathological examinations and immunohistochemical staining

Testicular and epididymis tissue specimens were fixed in 4% paraformaldehyde. Sections were prepared in accordance with standard procedures. Afterwards, the sections underwent staining with hematoxylin and eosin (H&E). Spermatogenesis and the thickness of the epithelium in all mice were analysed histologically. The Image J software from the National Institutes of Health in the United States was used to determine the number and thickness of tubules using images taken at 200× magnification. To block the activity of endogenous peroxidase, the sections were subjected to treatment with a 3% H_2_O_2_ solution for 3,3‐diaminobenzidine (DAB) staining and immunohistochemical analysis. To prevent binding to unspecified sites, a solution of 1% bovine albumin (BSA) in phosphate‐buffered saline (PBS) was applied for 1 h at room temperature. Following this, the sections were then exposed to anti‐GPR120 (Affinity, cat no. AF5219, 1:400) and anti‐12‐lipoxygenase (12‐LOX; Santa Cruz, cat no. sc‐365,194, 1:200) antibodies overnight at 4°C. Afterwards, the sections were cleansed and subsequently incubated with a secondary antibody, known as anti‐rabbit immunoglobulin G. DAB staining (Dako Cytomation, Carpinteria, CA) was used to visualize protein expression. Distilled water was used to halt the reaction, and after staining with haematoxylin and dehydration, the sections were mounted. The IX73 fluorescence microscope (Olympus Corporation, Shinjuku, Tokyo, Japan) was used to capture and digitize the images.

### 2.6 Immunofluorescence staining

The testicular tissues were treated with a 4% paraformaldehyde solution for fixation and then dehydrated in different concentrations of ethanol (70%, 90%, and 100%). Following embedding in paraffin blocks, the tissues underwent sectioning and subsequent rehydration, involving xylene for a duration of 30 min, followed by 5‐min intervals of 100%, 85%, and 75% ethanol. To perform immunostaining, the sections underwent permeabilization using 0.1% (v/v) Tween‐20. Following the addition of 3% (v/v) BSA to obstruct, the sections were subjected to incubation with anti‐DEAD‐box helicase 4 (DDX4/MVH) (Abcam, cat no. ab13840, 1:100), anti‐Lin28 (Abcam, cat no. ab46020, 1:200), and anti‐c‐kit (Abcam, cat no. ab32363, 1:100) antibodies for an extended period at 4°C. On the following day, the samples were exposed to secondary antibodies at ambient temperature in the absence of light for a duration of 50 min. Additionally, they were stained with Hoechst (diluted to 1:2000; Invitrogen) for 10 min at room temperature. In order to perform quantitative analysis of cells, the quantities of MVH^+^, Lin28^+^, and c‐kit^+^ cells in each cross‐section of the testis were tallied in five randomly selected fields per section. For each group, analysis was conducted on a minimum of three sections obtained from separate experiments. All images were obtained using an IX73 microscope.

### 2.7 TUNEL staining

Apoptotic cell detection was carried out using the One Step TUNEL Apoptosis Assay Kit (Beyotime Biotechnology, China) according to the guidelines provided by the manufacturer. In short, sections of testicular tissue were fixed in a 4% solution of paraformaldehyde (volume/volume) for 60 min, followed by a single wash with PBS. Subsequently, the tissue was exposed to PBS containing 0.1% Triton X‐100 for 2 min while kept on ice. After rinsing twice with PBS, the sections were treated with TUNEL fluorescent labelling solution and the enzyme TdT (terminal deoxynucleotidyl transferase) was introduced. Images were obtained with an Olympus microscope.

### 2.8 Transmission electron microscopy

Testicular specimens in culture dishes were fixed with a solution of PBS and 0.5% glutaraldehyde for a duration of 30 min. Following immobilization in a 2% agarose gel, the samples were left to incubate for 2 h in a water‐based solution containing 1% OsO4 and 1.5% hexacyanoferrate (II). Subsequently, they were washed with water and stored overnight at 4°C in a 1% aqueous uranyl acetate solution. Following a water wash and dehydration with acetone, the specimens were then immersed in Epon for embedding. Thin slices (60 nm) were placed on copper grids coated with formvar, stained with uranyl acetate and lead citrate, and examined under a transmission electron microscope (JEM‐1011, JEOL, Japan). Recordings of the images were made with a CCD camera mounted on the side.

### 2.9 Detection of lipid metabolites using ultra performance liquid chromatography‐tandem mass spectrometry (UPLC–MS/MS)

The lipid metabolites from testicular contents were determined via UPLC–MS/MS. In short, the UPLC (Shim‐pack UPLC Shimadzu CBM30A) and MS/MS (QTRAP 6500+) system was configured with a resolution of 30,000 in order to acquire UPLC–MS/MS data. The analysis was conducted in positive ion mode using a spray voltage of 5.5 kV and in negative ion mode using a spray voltage of −4.5 kV, while maintaining a capillary temperature of 500°C. The mass spectrometer was set to scan 50–1500 m/z. The nitrogen sheath and nitrogen auxiliary gas were adjusted to flow at rates of 30 and 10 L/min, respectively. Solvent A consisted of 0.04% acetic acid (Fisher Scientific) diluted with water (Millipore) in a volume‐to‐volume ratio, while solvent B was prepared by combining 0.04% acetic acid (Fisher Scientific) with acetonitrile (Fisher Scientific) in a volume‐to‐volume ratio. The rate of gradient flow was adjusted to 0.4 mL/min, while maintaining a column temperature of 40°C. The linear gradient was as follows: 5% B at 0 min, 95% B at 11.0 min, 95% B at 12.0 min, 5% B at 12.1 min, and 5% B at 14 min. To guarantee system stability, the QC samples were injected four times initially. For all analyses, a Waters ACQUITY UPLC HSS T3 C18 column measuring 100 mm × 2.1 mm with a particle size of 1.8 μm was utilized. The contents of lipid metabolites were detected using MetWare (http://www.metware.cn/).

### 2.10 Isolation and culture of primary mouse Sertoli cells and spermatogonia

Primary Sertoli cells from the testes of 20‐day‐old ICR mice (obtained from Beijing Vital River Laboratory Animal Technology Co., Ltd) were isolated for analysing cell function. The isolation was performed utilizing a two‐stage enzymatic digestion technique, as previously outlined.^23^ The cells were incubated for 2 days at 37°C under a 5% CO_2_ atmosphere using Dulbecco's modified Eagle's medium/Ham's nutrient mixture F12 (DMEM/F12; BasalMedia, Shanghai, China) supplemented with 10% foetal bovine serum (FBS; HAKATA, Shanghai, China). Next, the cells underwent treatment with 20 mM Tris (pH 7.4) for a duration of 3 min in order to eliminate germ cells. Subsequently, they were rinsed with PBS and then cultured until ready for utilization.

The method previously reported was used to isolate primary mouse spermatogonia.^24^ To summarize, mouse spermatogonia were obtained from 6‐day‐old mice. The collagenase IV (Sigma) was used to digest the testes at a concentration of 1 mg/mL for 15 min at 37°C. Then, 0.05% trypsin–EDTA (Thermo Fisher Scientific) was added to digest for 5 min at 37°C. The cells from the testicles were gathered and placed onto a culture dish coated with 0.1% (w/v) gelatin. After a period of 2 h, the detached cells were gathered and placed onto a different culture dish that had been coated with gelatin. Following three iterations, the detached cells were gathered and planted onto the feeder cells. Mouse embryonic fibroblasts (MEFs) were obtained from E12.5 embryos to create the feeder layer for spermatogonia. The StemPro‐34 SFM (Invitrogen, Carlsbad, CA) was used as the SSC medium, which was supplemented with StemPro Supplement (Invitrogen), 25 mg/mL insulin, 100 mg/mL transferrin, 60 mM putrescine, 30 nM sodium selenite, 6 mg/mL D‐(+)‐glucose, 30 μg/mL pyruvic acid, 1 μL/mL DL‐lactic acid (Sigma), 5 mg/mL BSA (ICN Biomedicals), 2 mM L‐glutamine, 5 × 10^−5^ M 2‐mercaptoethanol, MEM Vitamin Solution (Invitrogen), MEM non‐essential amino acids solution (Invitrogen), 10^−4^ M ascorbic acid, 10 μg/mL Dbiotin, 30 ng/mL β‐estradiol, and 60 ng/mL progesterone (Sigma). Primary mouse spermatogonia were placed onto a MEF feeder layer and incubated with SSC medium that contained 15 ng/mL GDNF and 10 ng/mL basic fibroblast growth factor at a temperature of 37°C and 5% CO_2_ for 7 days. It is important to regularly change the medium every 1–2 days.

### 2.11 Culture of TM4 cell line and GC‐1 cell line

Sertoli cells from TM4 were cultivated in regular DMEM/F12 supplemented with 10% FBS and 1% penicillin/streptomycin. GC‐1 cells were grown in DMEM/Basic medium containing 10% FBS, 1% penicillin/streptomycin, 0.1 mM non‐essential amino acids, 1 mM sodium pyruvate, and an additional 2 mM of L‐glutamine. Procell Life Science & Technology Co. Ltd. (Wu Hai, China) provided the TM4 cell line, a mouse Sertoli cell line that is available for purchase commercially. The commercially accessible SSC cell line, known as the GC‐1‐cell line, was acquired from KE LEI Biological Technology Co. Ltd. located in Shanghai, China. The cells were cultured in a CO_2_ incubator with 5% humidity at a temperature of 37°C.

### 2.12 Cell treatment

To uncover the mechanisms of 12‐HEPE, we utilized Sertoli cells and spermatogonia in our investigation. TM4 Sertoli cells and GC‐1 spermatogonia were treated with different concentrations of busulfan (0, 10^−6^, and 10^−4^ M), and the levels of reactive oxygen species (ROS) were measured. TM4 Sertoli cells and GC‐1 spermatogonia were treated with 10^−4^ M busulfan and harvested for the 5‐ethynyl‐2‐deoxyuridine (EdU) assay and RNA and protein extraction. TM4 Sertoli cells were treated with 15‐hydroxyeicosapentaenoic acid (15‐HEPE; cat no. GC40361, Glpbio) (1 μM), 12‐HEPE (1 μM), or 5‐(6)‐DiHETE (1 μM) (cat no. GC41119, Glpbio) and then with busulfan (10^−4^ M) for 24 h. Then, the cells were collected for protein extraction. TM4 Sertoli cells were treated with 12‐HEPE (1 μM) and collected at various time intervals (0, 6, 12, and 24 h) for western blotting and reverse transcription‐quantitative polymerase chain reaction (RT‐qPCR). In parallel, TM4 Sertoli cells were pre‐incubated with varying amounts of AH7614, an inhibitor of GPR120 (0, 10, or 25 μM) for 2 h and then with 12‐HEPE (1 μM) for an additional 24 h. Following this, the cells were collected for protein extraction. TM4 Sertoli cells were subjected to treatment with 12‐HEPE (1 μM) and collected at various time intervals (0, 5, 10, 30, 60, and 90 min) to analyse the levels of total and phosphorylated ERK1/2 MAPK. Afterwards, TM4 Sertoli cells were treated with different concentrations of 12‐HEPE (0 or 1 μM) and different concentrations of AH7614 (0 or 25 mM) or siRNA‐GPR120 for different durations (0, 60, and 90 min). Prior to western blot analysis of BMP4 levels, TM4 and primary mouse Sertoli cells underwent pre‐incubation with the p‐ERK inhibitor PD98059 (CAS 167869–21‐8, Selleck). Subsequently, they were exposed to varying concentrations of PD98059 (0, 2, or 10 μM) for a duration of 2 h, followed by treatment with 12‐HEPE (1 μM) for an additional 24 h.

### 2.13 Transfection of siRNAs

GenePharma (Shanghai, China) designed and synthesized mouse GPR120‐specific siRNAs (siRNA‐GPR120) as well as a non‐specific siRNA (scrambled siRNA; negative control). To knockdown the expression of GPR120, cells were transfected with siRNA‐GPR120 for 6 h before the indicated treatments. Cells were transfected with siRNA‐GPR120 for a duration of 6 h prior to the specified treatments in order to suppress the expression of GPR120. The transfection process was carried out by utilizing lipofectamine 3000 reagent (cat no. L3000015; Invitrogen) in accordance with the guidelines provided by the manufacturer. Table S2 displays the sequences of siRNA‐GPR120.

### 2.14 Co‐culture experiment and conditioned medium treatment

After transfecting TM4 or primary mouse Sertoli cells with siRNA‐GPR120 for 6 h to knockdown the expression of GPR120, they were treated with both 1 μM 12‐HEPE and 10^−4^ M busulfan for 24 h. GC‐1 or primary mouse spermatogonia were placed in the bottom compartment of a transwell plate (0.4 μm) at a density of 1 × 10^5^/well. The next day, the TM4 or primary mouse Sertoli cells (donor cells) were added to the top compartment of the transwell plate at same density. After incubation for 24 h, the cells were collected to extract proteins. The medium of the Sertoli cells treated as described above was diluted 1:1500 and used to induce spermatogonia differentiation and proliferation. The spermatogonia were harvested for protein extraction.

### 2.15 Assessment of ROS levels and EdU incorporation

In addition, cell proliferation was evaluated using a BeyoClick™ Edu Cell Proliferation Kit with Alexa Fluor 594 (cat no. C0071 L; Beyotime). The procedure was performed according to the manufacturer's instructions. Images were taken with an IX73 microscope and digitized. The fluorescent dye 2,7‐dichlorodihydrofluorescein diacetate (H_2_DCFDA, KeyGEN BioTECH) was utilized to examine the levels of intracellular ROS. In short, the cells that were treated were placed in DMEM with a concentration of 10 μM H_2_DCFDA and incubated for 30 min at a temperature of 37°C. In addition, the proliferation of cells was assessed by employing a BeyoClick™ Edu Cell Proliferation Kit containing Alexa Fluor 594 (cat no. C0071 L; Beyotime). Images were taken with an IX73 microscope and digitized.

### 2.16 Cell immunofluorescence

To perform immunofluorescence staining, cells seeded in 24‐well plates were treated with 0.1% (v/v) Triton X‐100 and fixed for a duration of 20 min. Afterwards, cells were rinsed using PBS on three occasions, followed by blocking with 10% goat serum in neutral PBS for 30 min at room temperature. Subsequently, they were exposed to a rabbit polyclonal anti‐GPR120 antibody (Affinity, cat no. AF5219, 1:400) and incubated overnight at 4°C. Following three washes with PBS, the cells were then exposed to the suitable secondary antibodies for 1 h. Additionally, the nuclei were stained with Hoechst (diluted 1:2000; Invitrogen) for visualization.

### 2.17 RNA extraction and RT‐qPCR

The cells were used to extract total RNA using a Total RNA Isolation Kit (BEI‐BEI Biotech). To analyse mRNA, cDNA was generated from total RNA by utilizing the reverse transcriptase PrimeScript® RT Master Mix (TaKaRa). The cDNA was employed for qPCR utilizing a Roche Light Cycler 96 Real‐time PCR System manufactured by Roche Diagnostics located in Switzerland. SYBR Green Premix Pro Taq HS qPCR tracking kit (AG11733) was purchased from Accurate Biotechnology Company (Changsha, China). The comparative ∆∆Ct method was utilized to calculate the relative expression with GAPDH as internal control. Table S3 contains the primer sequences.

### 2.18 Protein extraction and western blotting

Samples of tissue and cells from the testis were rinsed with ice‐cold 1× PBS and then immediately lysed in ice‐cold RIPA (Sigma), supplemented with 1 mM PMSF (Beyotime) and 1 mM protease inhibitor cocktail (Sigma). Protein concentrations were measured using the BCA test (Thermo Fisher Scientific). Protein with same concentrations were mixed with 1× sodium dodecyl sulfate polyacrylamide gel electrophoresis loading buffer, heated at 95°C for 10 min, and subsequently placed onto Bio–Rad's Criterion TGX 4%–20% gels. After transferring, the proteins were placed onto polyvinylidene fluoride membranes (Bio‐Rad), and the quality of the transfer was evaluated using Ponceau staining. The membranes were obstructed for 1 h at room temperature in antibody diluent (NCMbiotech). Subsequently, they were incubated overnight at 4°C with the subsequent antibodies, which were diluted in 5% BSA: anti‐PLZF (Santa Cruz, cat no. sc‐28,319, 1:200), anti‐Lin28 (1:1000), anti‐c‐kit (1:750), anti‐Stra8 (Abcam, cat no. ab49602, 1:1000), anti‐SYCP3 (Abcam, cat no. ab15093, 1:1000), anti‐BMP4 (Abcam, cat no. ab39973, 1:1000), anti‐Rdh10 (Proteintech, cat no. 14644‐1‐AP, 1:1000), anti‐GPR120 (1:1000), anti‐phospho‐ERK1/2 (Cell Signaling, cat no. 4370, 1:2000), anti‐ERK1/2 (Cell Signaling, cat no. 4695, 1:1000), anti‐Vinculin (Proteintech, cat no. 66305‐1‐lg, 1:2000), and anti‐β‐actin (Proteintech, cat no. 66009‐1‐lg, 1:3000). The membranes were rinsed three times for 5 min using 1× tris buffered saline with tween‐20 (TBST) and subsequently exposed to the respective secondary horseradish peroxidase‐conjugated antibody, which was diluted in 1× TBST, for 1 h at room temperature. The membranes underwent three washes with 1× TBST and were subsequently treated with ECL solution (Tanon Science and Technology) for development. The target band intensities were quantified using ImageJ software to calculate the relative protein expression.

### 2.19 Statistical analysis

GraphPad Prism version 8 was utilized to conduct statistical analysis. The normality of the data was analysed first, and then statistical tests were performed. The Student's *t*‐test, the Mann–Whitney test, and one‐way analysis of variance followed by Tukey's post hoc test were employed. Metabolites that were significantly regulated between groups were identified using criteria including variable information processing (VIP) ≥ 1, *p*‐value < 0.05, and absolute Log_2_FC ≥1. The orthogonal partial least squares‐discriminant analysis results included score plots and permutation plots generated using the R package ropls, from which VIP values were extracted. The *p*‐value for the significance of each metabolite was calculated using the Mann–Whitney test. The mean ± standard error of the mean represents all the remaining data. The figures represent *p*‐values, which indicate the following: not significant [ns], *p* > 0.05; *p < 0.05; **p < 0.01; ***p < 0.001.

## 3 RESULTS

### 3.1 Patients with severe dyszoospermia have a notably reduced the omega‐3/omega‐6 ratio in seminal plasma

**FIGURE 1:**
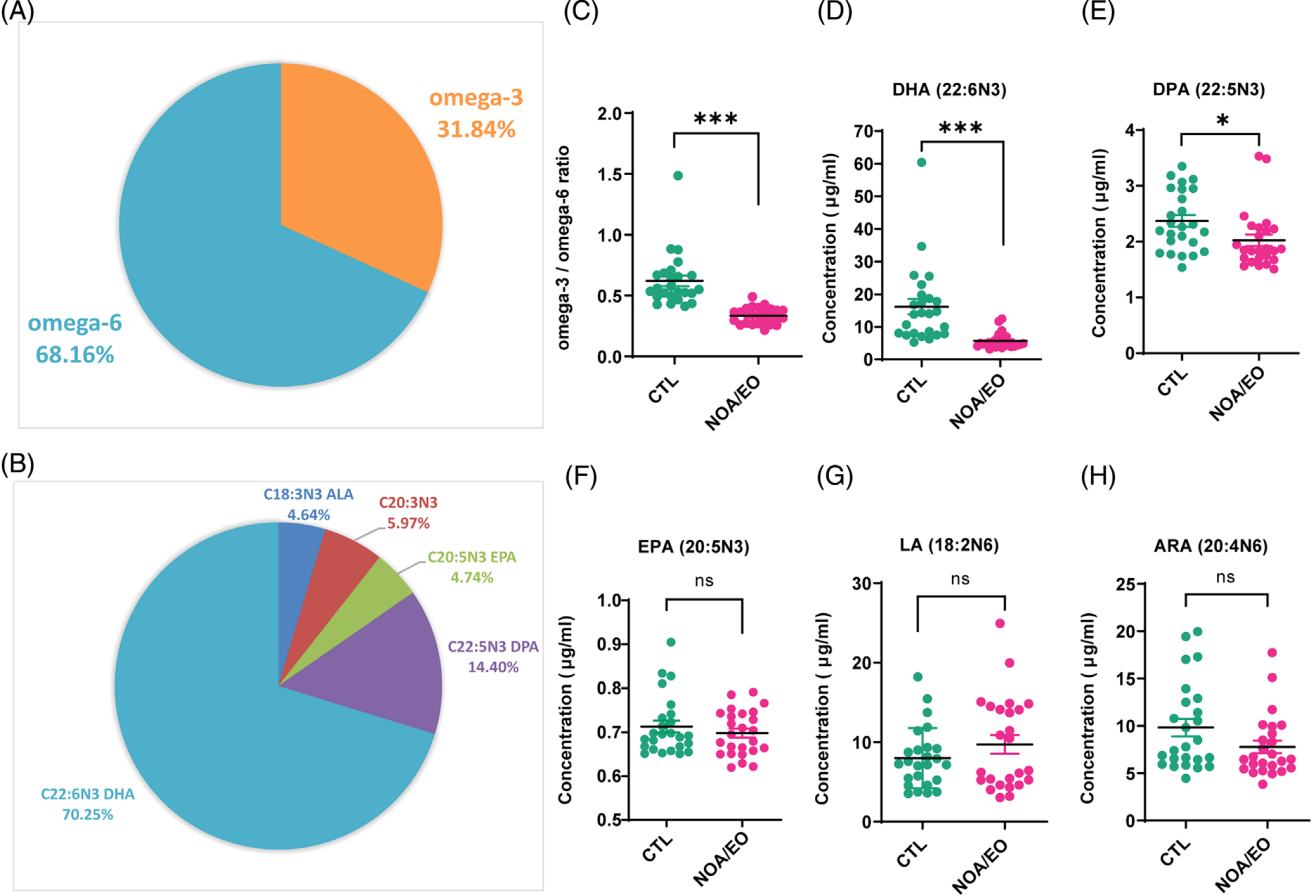
The omega‐3/omega‐6 ratio in seminal plasma is significantly lower in patients with severe dyszoospermia. The proportions of polyunsaturated fatty acid (PUFA) constitution (A) and different components of omega‐3 (B) were measured. The omega‐3/omega‐6 ratio (F), docosahexaenoic acid docosahexaenoic acid (DHA) (C), docosapentaenoic acid (DPA) (D), eicosapentaenoic acid (EPA) (E), linoleic acid (LA) (G), and arachidonic acid (ARA) (H) levels in the seminal plasma were measured by gas chromatography coupled with mass spectrometry. The study included a group of healthy controls (*n* = 25) and two groups of patients: one with non‐obstructive azoospermia (NOA, *n* = 21) and the other with extreme oligospermia (EO, *n* = 4). Compared with the healthy control group: *p < 0.05; **p < 0.01; ***p < 0.001; ns, *p* > 0.05.

Our clinical analysis focused on defect in spermatogenesis, specifically NOA and EO, in order to comprehend the potential correlation between the constitution of PUFAs and this condition. In order to examine analyse free fatty acid levels in clinical samples, we collected seminal plasma from a total of 50 individuals, including 21 patients with NOA, 4 patients with EO, and 25 healthy controls. Using GC/MS, we quantified the concentrations of 39 free fatty acids (C6–C24) with medium and long chains. We focused on PUFA levels in seminal plasma and found that omega‐3 PUFAs accounted for 31.84% of the total PUFA content (Figure 1A). As expected, DHA was the major component, accounting for 70.25% of the seminal plasma omega‐3 content (Figure 1B). The seminal plasma omega‐3/omega‐6 ratio was reduced in patients with severe dyszoospermia (NOA and EO) compared with healthy controls, and this reduction was accompanied by a significant decrease in DHA and docosapentaenoic acid (DPA) levels but not a dramatic decrease in EPA levels (Figure 1C–F). However, patients with severe dyszoospermia (NOA and EO) did not exhibit any notable change in seminal plasma omega‐6 levels of linoleic acid (LA) and arachidonic acid (ARA; Figure 1G,H). These clinical metabonomic results indicate a relationship between deficiency of omega‐3 PUFAs and disturbed spermatogenesis.

### 3.2 Dietary omega‐3 rescues busulfan disrupted spermatogenesis in vivo

**FIGURE 2:**
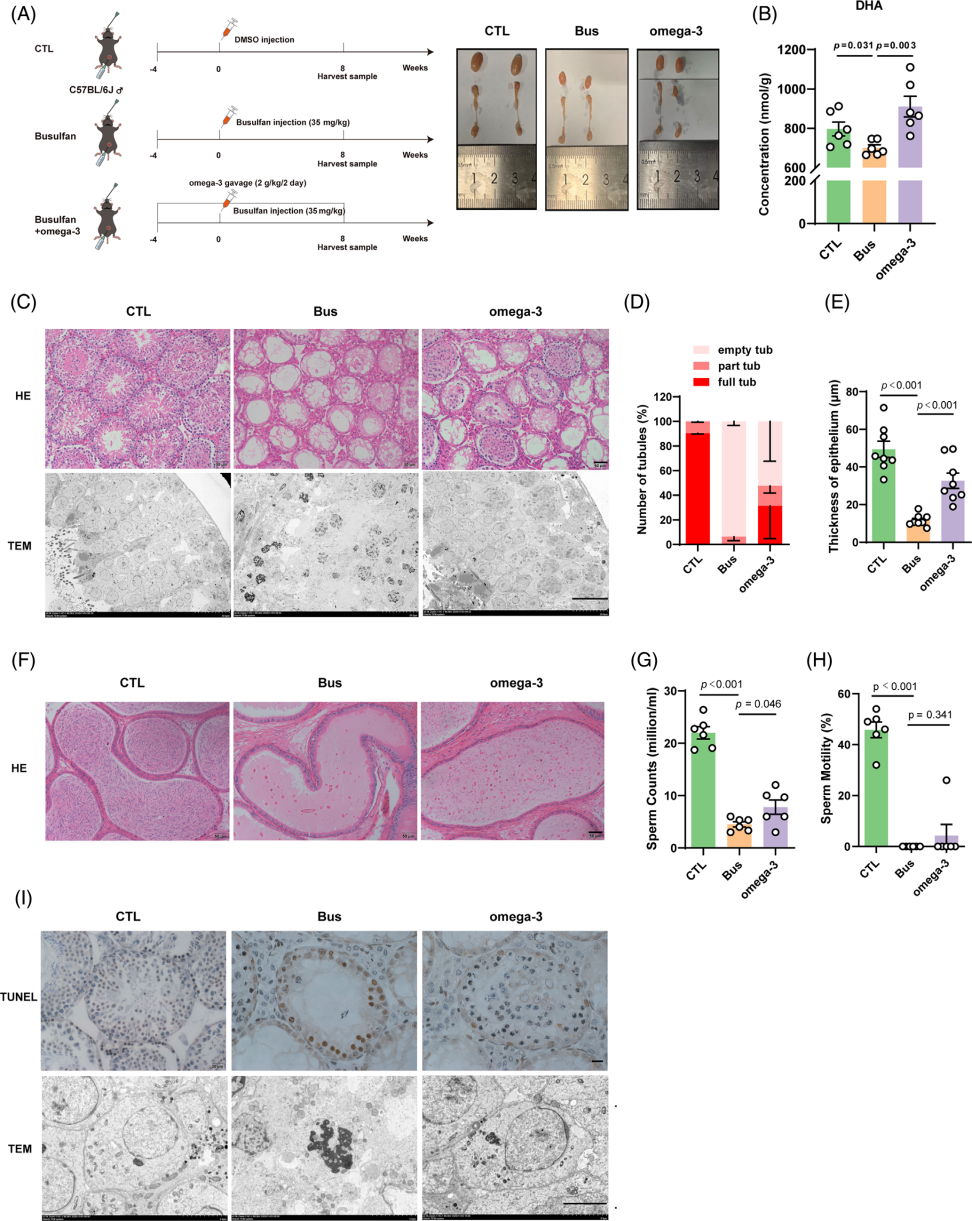
Dietary omega‐3 protects busulfan disrupted spermatogenesis in vivo. (A) Diagram of the animal experiments and images of testis morphology. (B) The docosahexaenoic acid (DHA) levels in testicular tissue from each group (*n* = 6). (C) Representative hematoxylin and eosin (H&E) staining images and transmission electron microscopy images of testicular cross‐sections from each group (*n* = 8 per group; scale bar, 50 μm). Testicular cross‐sections were examined using representative H&E staining and transmission electron microscopy from every group (*n* = 8; scale bar, 50 micrometres). Comparison between the model mice and control mice involves assessing the quantity of seminiferous tubules exhibiting empty, partial or full spermatogenesis (D) and tubule thickness (E). (F) Representative H&E staining images of caudal epididymis cross‐sections from each group (*n* = 8; scale bar, 50 μm). The sperm count (G) and motility (H) of the model mice were assessed and compared with those of control mice. I TUNEL staining of testicular spermatogenic tubules from each group and the morphology of spermatogonial cells under projective electron microscopy (*n* = 8; scale bar, 20 μm).

Low sperm counts and inadequate sperm motility are the primary factors linked to male fertility. Our assessment aimed to determine whether omega‐3 supplementation can restore spermatogenesis and improve sperm production and maturation in mice. The experimental procedure was illustrated in the schematic diagram (Figure 2A), pubertal or adult male mice received oral gavage of omega‐3 and also given busulfan injection as a single dose. The weight ratios for the testis and epididymis of omega‐3‐treated mice were not significantly different from those of busulfan‐treated mice for 8 weeks (Figure S1). The levels of sex hormones also showed no change between these two groups (Figure S2). The DHA level in the testicular tissue of mice treated with busulfan was lower compared with that of control mice, which was consistent with a observably decrease in seminal plasma DHA in patients with severe dyszoospermia. Importantly, the omega‐3‐treated mice showed a remarkably restoration of DHA level in their testicular tissue, which rescued the decreased DHA level due to the busulfan treatment (Figure 2B).

The omega‐3‐treated mice showed a significant rise in the quantity of tubules exhibiting both partial and complete spermatogenesis and a dramatical reduction in the quantity of empty spermatogenesis tubules, in contrast to the mice treated with busulfan (Figure 2C,D). The ultrastructural characteristics of the seminiferous tubule epithelium were assessed. The structural arrangement of seminiferous tubule epithelium was partially restored in mice treated with omega‐3, in contrast to those in busulfan group(Figure 2C). In testicular tissue of mice treated with omega‐3, the thickness of the seminiferous tubule epithelium, which indicates spermatogenesis, also showed a dramatical increase when compared with those of mice treated with busulfan (Figure 2E). H&E staining showed omega‐3 treatment resulted in a notable rise in the quantity of sperm in the cauda epididymidis of mice, in contrast to the mice treated with busulfan. (Figure 2F). Notably, the omega‐3‐treated mice exhibited a notably higher sperm count compared with the busulfan‐treated mice (Figure 2G). Sperm motility is essential for sperm maturation. In omega‐3‐treated mice, the motility of epididymal sperm was greater compared with busulfan‐treated mice, but the difference was not significant. (Figure 2H). TUNEL staining indicated a reduction in the quantity of apoptotic spermatogenic cells in mice treated with omega‐3, in contrast to mice treated with busulfan (Figure 2I).The ultrastructural characteristics of the spermatogenic cell nuclei were assessed. The spermatogenic cells of omega‐3‐treated mice showed partial restoration of the nuclear structure when compared with the busulfan‐treated mice (Figure 2I). The spermatogenic cells of omega‐3‐treated mice showed partial restoration of the nuclear structure when compared with the busulfan‐treated mice (Figure 2I). In total, our results demonstrate that omega‐3 therapy can expedite the recovery of spermatogenesis following busulfan treatment.

### 3.3 Dietary omega‐3 increases spermatogonia proliferation and differentiation in vivo

**FIGURE 3:**
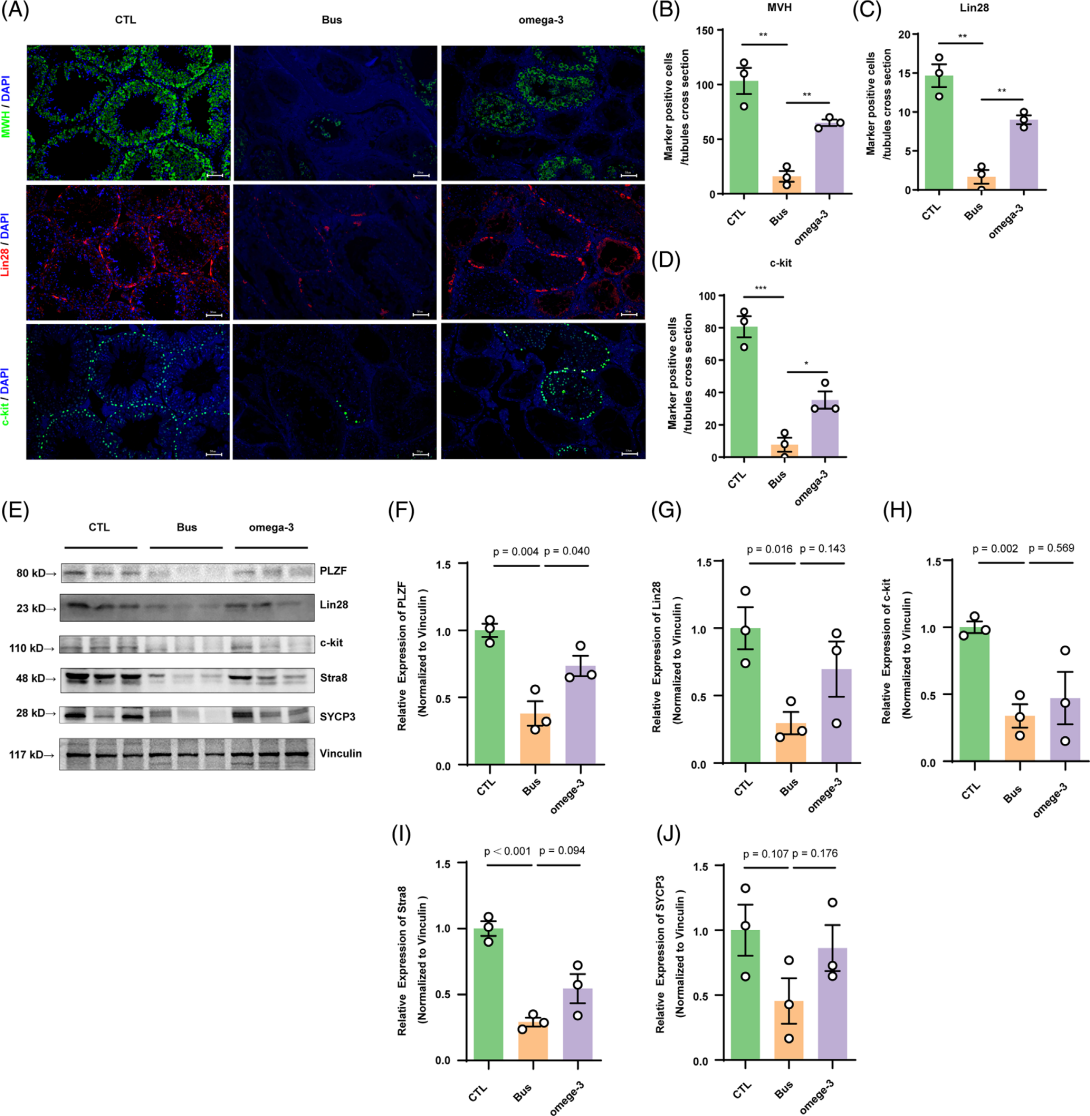
Dietary omega‐3 increases busulfan disrupted spermatogonia proliferation and differentiation in vivo. (A) Immunohistochemical staining indicated that Lin28 (red) was expressed in undifferentiated spermatogonia, and c‐kit (green) was found in differentiated spermatogonia in all groups (scale bar, 50 μm). (B–D) The count of MVH^+^, Lin28^+^, or c‐kit^+^ cells per cross‐section of tubules (*n* = 3). Compared with the control group: *p < 0.05; **p < 0.01; ***p < 0.001. (E) The protein expression of PLZF, Lin28, c‐kit, Stra8 and SYCP3 in each group (*n* = 3) was assessed by western blotting and compared them to the protein levels in control mice. (F–J) The quantification of germ cell markers' protein expression was quantified by normalizing the levels of these proteins to the level of the internal control vinculin. The data are displayed as the mean ± SEM from a minimum of three separate trials.

We further define if the therapeutical benefits of omega‐3 were dependent on improving endogenous population of spermatogonia. Testis sections were subjected to immunofluorescence for MVH to detect germ cells (Figure 3A,B). In mice treated with busulfan, there was a low occurrence of MVH‐positive cells found within seminiferous tubules, suggesting that busulfan caused long‐lasting or irreversible damage to spermatogenesis. In contrast, there was a dramatical rise in the quantity of MVH‐positive cells observed in mice treated with omega‐3. In addition, we investigated the presence of Lin28, an indicator of undifferentiated spermatogonia, and c‐kit, an indicator of differentiated spermatogonia, in all mouse groups (Figure 3A,C,D). Omega‐3 treatment resulted in a higher quantity of Lin28‐ and c‐Kit‐positive cells in the testicular cross‐sections compared with those from mice treated with busulfan. Furthermore, in comparison to busulfan, omega‐3 stimulated the production of proteins associated with undifferentiated spermatogonia (PLZF, Lin28), differentiated spermatogonia (c‐kit, Stra8), and spermatocytes (SYCP3; Figure 3E–J). Collectively, these results suggest that omega‐3 enhances the proliferation and differentiation of spermatogonia following busulfan treatment, ultimately leading to the restoration of spermatogenesis.

### 3.4 Dietary omega‐3 changes the levels of lipid metabolites in testicular tissue

**FIGURE 4:**
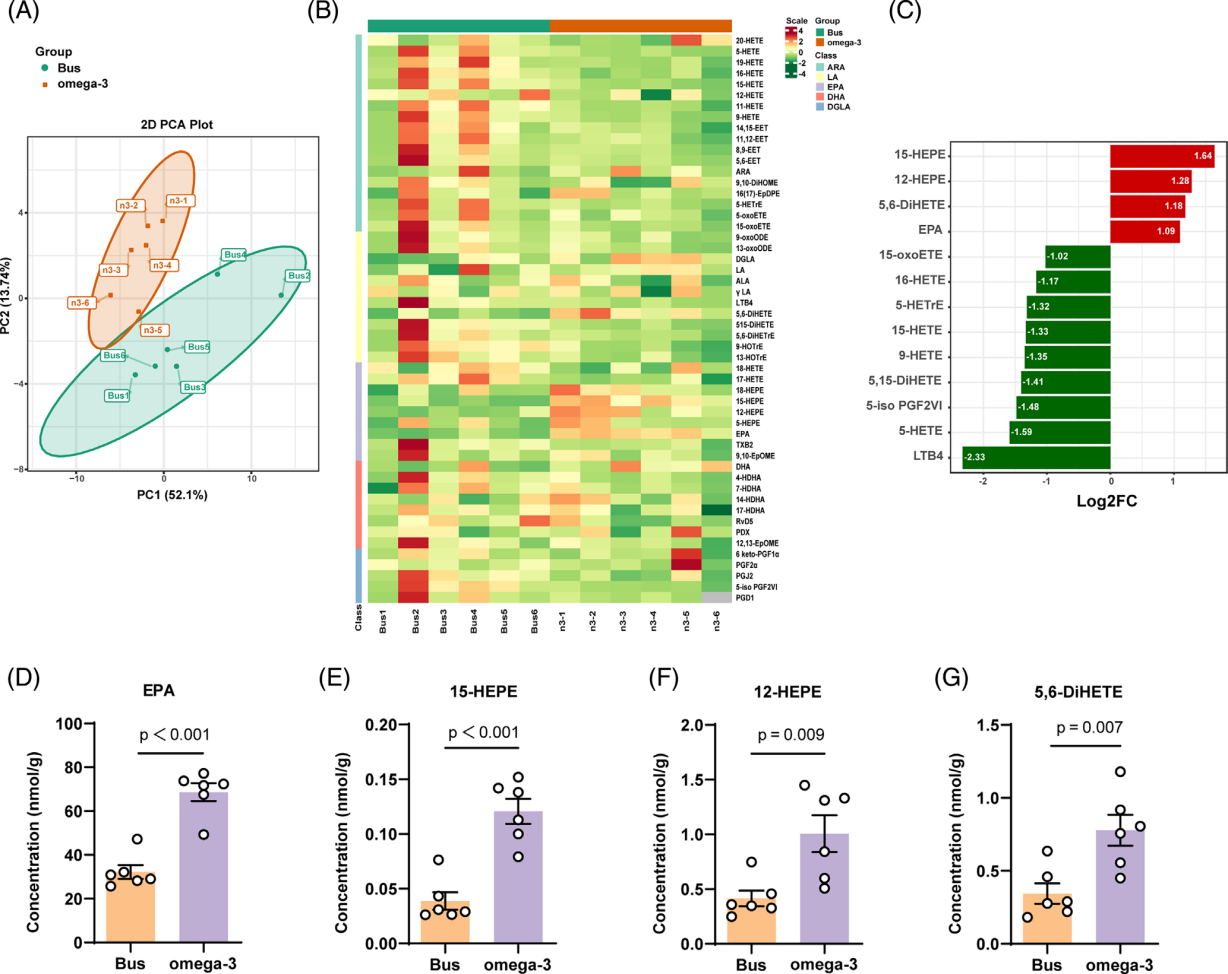
Dietary omega‐3 alters lipid metabolite levels in testicular tissue. (A) Principal component analysis (PCA) of metabonomic data from testicular tissue samples from the two groups (*n* = 6 per group). (B) Heatmap showing eicosanoid levels in testicular tissue samples from mice treated with busulfan and omega‐3 PUFAs (*n* = 6 per group). (C) Chart showing the fold change (FC) in the levels of metabolic products that showed an FC (log2) of at least two between the busulfan‐ and omega‐3‐treated groups. The red bars indicate compounds that were found at higher concentrations in omega‐3‐treated group, while the green bars indicate compounds that were found at lower concentrations in the omega‐3‐treated group. (D‐G) Quantitation of lipid metabolite levels in testicular tissue. The levels of eicosapentaenoic acid (EPA), 15‐hydroxyeicosapentaenoic acid (15‐HEPE), 12‐hydroxyeicosapentaenoic acid (12‐HEPE) and 5,6‐dihydroxy‐eicosatrienoic acid (5,6‐DiHETE) in testicular tissue from the omega‐3‐treated group were markedly elevated compared with the busulfan‐treated groups (*n* = 6). The data are displayed as the mean ± SEM.

To further explore the mechanisms that contribute to the beneficial impact of omega‐3 on spermatogenesis, we examined the change in the levels of lipid metabolites in testicular tissue between the omega‐3 and busulfan‐treated groups. The metabolomics data from the two groups underwent principal component analysis (Figure 4A). The levels of total metabolites in mouse testicular tissue are shown in the heatmap (Figure 4B). According to the bar graph depicting differential metabolites, the omega‐3‐treated group exhibited significantly elevated levels of four metabolites, and reduced levels of nine metabolites compared with the busulfan‐treated group. These changes in concentration were indicated by the fold change (FC) (log_2_FC ≥1 or log_2_FC ≤ −1) (Figure 4C).

Among the metabolites of EPA, the levels of 15‐HEPE, 12‐HEPE, and 5,6‐dihydroxy‐eicosatrienoic acid (5,6‐DiHETE) were significantly higher in the testicular tissue of mice treated with omega‐3 (Figure 4D–G). In contrast, among the metabolites of ARA, the levels of 5‐HETE, 9‐HETE, 15‐HETE, 16‐HETE, 5‐HETrE, 5‐iso PGF2VI, 5,15s‐DiHETE, 15‐oxoETE, and LTB4 were significantly lower in the testicular tissue of mice treated with omega‐3 (Figure S3). These metabonomic data provide evidence that dietary omega‐3 increases the levels of EPA and its metabolites in testicular tissue.

### 3.5 12‐HEPE enhances BMP4 expression in Sertoli cells to rescue spermatogonia proliferation and differentiation

**FIGURE 5:**
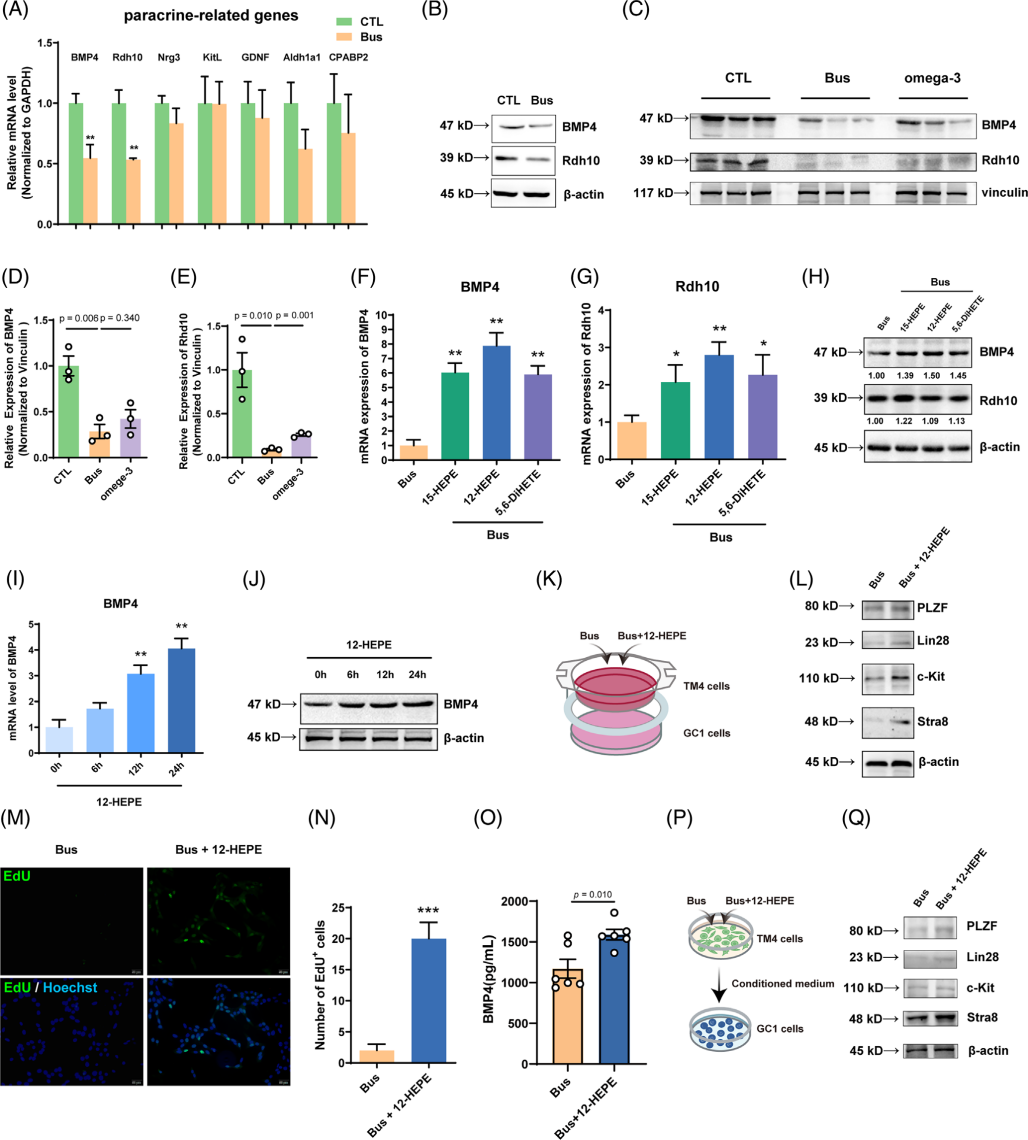
12‐Hydroxyeicosapentaenoic acid (12‐HEPE) enhances bone morphogenic protein 4 (BMP4) expression in Sertoli cells to rescue spermatogonia proliferation and differentiation. (A,B) After subjecting TM4 cells to a 24‐h treatment of 10^−4^ M busulfan, the expression of paracrine factors was assessed using reverse transcription‐quantitative polymerase chain reaction (RT‐qPCR) for mRNA and western blotting for protein. Compared with the control group: **p < 0.01. (C–E) In omega‐3‐treated mice, BMP4 and Rdh10 protein expression was quantified by western blotting. (F–H) TM4 cells were treated with 15‐hydroxyeicosapentaenoic acid (15‐HEPE), 12‐HEPE, or 5‐(6)‐DiHETE and then with busulfan for 24 h, and the mRNA and protein levels of BMP4 and Rdh10 were examined by RT‐qPCR and western blotting, respectively. TM4 cells were exposed to 15‐HEPE, 12‐HEPE, or 5,6‐dihydroxy‐eicosatrienoic acid (5,6‐DiHETE) followed by busulfan treatment for 24 h. The expression of BMP4 and Rdh10 at mRNA and protein levels was assessed using RT‐qPCR and western blotting. (I,J) TM4 cells were treated with 12‐HEPE at various time points and the expression of BMP4 and Rdh10 at mRNA and protein levels were assessed using qRT‐PCR and western blotting. (K) Schematic of the co‐culture system including GC‐1 cells and TM4 cells treated with busulfan or Bus+12‐HEPE. (L) The protein expression of germ cell markers were assessed using western blotting in GC‐1 cells. (M) The 5‐ethynyl‐2‐deoxyuridine (EdU) incorporation assay was used to assess GC‐1 cell proliferation (scale bar, 50 μm). (N) The count of cells labelled with EdU in each group. In comparison to the group that received only busulfan: ***p < 0.001. (O) TM4 cells were exposed to 10^−4^ M busulfan alone or in combination with 12‐HEPE. The ELISA method was used to evaluate the secretion of BMP4 in the culture medium. (P) Schematic of the protocol used to treat GC‐1 cells treated with conditioned medium from TM4 cells pre‐treated with treated with busulfan or Bus+12‐HEPE for 24 h. (Q) The protein levels of germ cell markers were assessed using western blotting in GC‐1 cells. The data are displayed as the mean ± SEM from a minimum of three separate trials.

We investigated how the increase in the levels of lipid metabolites contributes to spermatogonia proliferation and differentiation. A DCFH‐DA assay was employed to measure the level of ROS following busulfan at concentrations of 10^−6^ and 10^−4^ M using immunofluorescence. After the administration of busulfan, there was a significant rise in ROS levels detected in GC‐1 spg cells (a spermatogonia cell line) but not in a TM4 cells (a Sertoli cell line) within 30 min (Figure 4A). Thus, the damaging effects of busulfan‐induced oxidative stress are greater on spermatogonia compared with Sertoli cells. Interestingly, the inhibition of proliferation and differentiation‐ associated genes, including *PLZF, Lin28, c‐kit, Stra8, Rhd10, Aldh1a2*, and *Crabp1*, was observed in GC‐1 spg cells when exposed to 200 mM hydrogen peroxide (H_2_O_2_) or 10^−4^ M busulfan, in contrast to untreated control GC‐1 spg cells (Figure 4B). Consequently, we investigated the impact of busulfan on the proliferation of GC‐1 spg cells. The number of EdU‐incorporating cells was decreased after treatment with 10^−4^ M busulfan (Figure 4C,D). Furthermore, the mRNA and protein expression of paracrine‐associated genes, including *BMP4* and *Rhd10*, was inhibited in TM4 cells treated with 10^−4^ M busulfan compared with untreated control TM4 cells (Figure 5A,B). Additionally, compared with busulfan, omega‐3 facilitated the protein expression of BMP4 and Rhd10 in testicular tissue of mice (Figure 5C–E).

We investigated which of the lipid metabolites that showed an increase in concentration mainly contribute to spermatogonia proliferation and differentiation. In TM4 cells, the levels of *BMP4* and *Rhd10* mRNA expression were notably increased treated with 100 ng/mL 15‐HEPE, 12‐HEPE, and 5,6‐DiHETE compared with TM4 cells treated with 10^−4^ M busulfan (Figure 5F,G). Furthermore, the levels of protein expression for BMP4 were higher in TM4 cells treated with 100 ng/mL 12‐HEPE than those treated with 15‐HEPE or 5,6‐DiHETE compared with TM4 cells treated with 10–4 M busulfan (Figure 5H), so we chose 12‐HEPE as representative metabolite for the following study. Treatment with 100 ng/mL 12‐HEPE significantly induced BMP4 mRNA and protein expression after 12 and 24 h, respectively (Figure 5I,J). The placement of TM4 or primary Sertoli cells treated with 12‐HEPE in the upper chamber of a Transwell system, while GC‐1 or primary spermatogonia were seeded in the lower chamber (Figures 5K and S8A). The expression levels of proliferation and differentiation‐related proteins, including PLZF, Lin28, c‐kit, and Stra8, were significantly elevated in GC‐1 or primary spermatogonia treated with 100 ng/mL 12‐HEPE compared with those treated with 10^−4^ M busulfan (Figures 5L and S8A). Moreover, the number of EdU‐incorporating GC‐1 spg cells was increased after treatment of transwell system with 12‐HEPE (Figure 5M,N). The levels of proteins related to proliferation and differentiation in GC‐1 spg cells cultured alone did not show an increase when exposed to 12‐HEPE (Figure S5). 12‐HEPE significantly induced the secretion of BMP4 into the culture medium of TM4 cells (Figure 5O). The results of the conditioned medium experiment were in accordance with the results of the co‐culture experiment in this study (Figure 5P,Q). In summary, our findings reveal that 12‐HEPE mainly enhances BMP4 expression in Sertoli cells, leading to spermatogonia proliferation and differentiation in vitro.

### 3.6 12‐HEPE regulates BMP4 expression in Sertoli cells via the GPR120‐ERK signalling pathway

**FIGURE 6:**
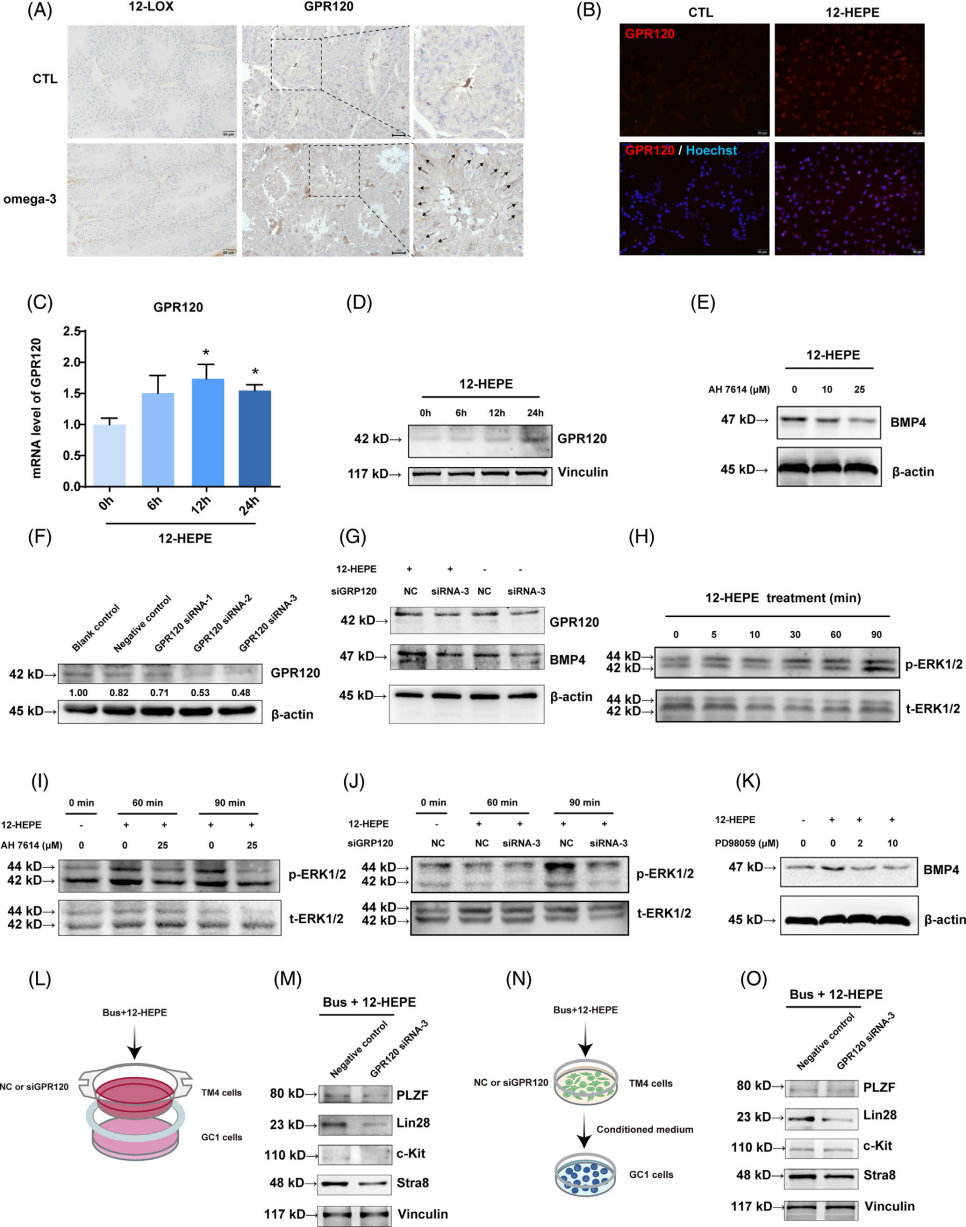
12‐Hydroxyeicosapentaenoic acid (12‐HEPE) regulates bone morphogenic protein 4 (BMP4) expression in Sertoli cells via the G protein‐coupled receptor 120 (GPR120)‐ERK signalling pathway. (A) GPR120 and 12‐lipoxygenase (12‐LOX) protein expression was assessed by immunohistochemistry. The arrows indicate positive staining (scale bar, 50 μm). (B) After a 24‐h treatment of TM4 cells with 12‐HEPE, the immunofluorescence analysis was conducted to the protein expression of GPR120 (red) (scale bar, 50 μm). (C,D) TM4 cells were exposed to 12‐HEPE for 0, 6, 12, or 24 h. The expression of GPR120 was assessed using reverse transcription‐quantitative polymerase chain reaction for mRNA and western blotting for protein levels. (E) TM4 cells were pretreated with different concentrations of AH7614 for a duration of 2 h to assess the expression of BMP4 protein. (F) After transfecting with three distinct siRNAs that targeted GPR120, blank control, or negative control (scrambled) siRNA for a duration of 24 h, and GPR120 protein expression was measured. (G) The cells were transfected with siRNA‐GPR120‐3 or scrambled siRNA and subsequently exposed to 12‐HEPE for 24 h. GPR120 and BMP4 protein expression was assessed. (H) TM4 cells were treated with 12‐HEPE for different durations, and the phosphorylated ERK1/2 levels were assessed. (I,J) TM4 cells were pretreated with AH7614 (a GPR120 inhibitor) or transfected with siRNA‐GPR120‐3 and then treated with 12‐HEPE for different durations. The phosphorylated ERK1/2 levels were assessed. (K) TM4 cells were pretreated with various concentrations of PD98059 (a p‐ERK inhibitor) for 2 h and then treated with 12‐HEPE for another 24 h. Then, BMP4 protein expression was evaluated by western blotting. TM4 cells were pre‐exposed to different doses of PD98059, an inhibitor of p‐ERK, for a duration of 2 h. Subsequently, BMP4 protein expression was assessed. (L) Schematic of the co‐culture system including GC‐1 and TM4 cells transfected with siRNA‐GPR120‐3 or scrambled siRNA. Afterward, they were treated with busulfan and 12‐HEPE for 24 h. (M) The germ cell markers protein expression was evaluated and compared with negative control group. (N) Schematic of the protocol used to treat GC‐1 cells with conditioned medium from TM4 cells transfected with siRNA‐GPR120‐3 or scrambled siRNA and then treated with busulfan and 12‐HEPE for a duration of 24 h. (O) The germ cell markers protein expression was evaluated and compared with negative control group. The data are displayed as the mean ± SEM from a minimum of three separate trials.

In order to resolve the mechanism underlying the regulatory effect of 12‐HEPE on BMP4 expression, we assessed the localization of 12‐LOX, which converts EPA to 12‐HEPE, and GPR120, a 12‐HEPE receptor/sensor, in the mouse testes. Interestingly, the immunohistochemical staining revealed the accumulation of GPR120 in the Sertoli cells in omega‐3‐treated mice, while 12‐LOX was not visibly localized to and showed no changes in concentration in the testes (Figure 6A). These results suggest that 12‐HEPE might be not produced in the testis but interacts with Sertoli cells. Furthermore, treatment with 100 ng/mL 12‐HEPE for 12 and 24 h significantly elevated the mRNA and protein expression of GPR120, respectively, compared with baseline levels (Figure 6B–D).

We conducted additional investigations into the function of GPR120 in the 12‐HEPE‐mediated increase in BMP4 expression in Sertoli cells. We used a chemical antagonist of GPR120 (AH7614; pretreatment with 0, 10, or 25 μM for 2 h pretreatment) and analysed BMP4 expression. The protein levels of BMP4 were dramatically reduced with increasing concentrations of AH7614 treatment compared with 12‐HEPE treatment alone (Figure 6E). In addition, Sertoli cells were transfected with three duplexes of GPR120 siRNA and a scrambled siRNA. Among the three duplexes, it was observed that siRNA3 exhibited the most effective silencing effect (Figure 6F). Thus, siRNA3 was used in subsequent experiments. However, the increase in BMP4 expression induced by 12‐HEPE was evidently decreased via transfection with GPR120 siRNA3 compared with transfection with scrambled siRNA in TM4 and primary Sertoli cells (Figures 6G and S8B). Next, we investigated whether activation of ERK1/2 is involved in 12‐HEPE‐stimulated BMP4 expression. 12‐HEPE (100 ng/mL) increased ERK1/2 phosphorylation as early as 60 min after treatment, then ERK1/2 phosphorylation remained increased compared with initial levels after 90 min (Figure 6H). In addition, the increase in p‐ERK1/2 expression induced by 12‐HEPE was hindered by AH7614 and siRNA‐GPR120 (Figure 6I,J). The effect of 12‐HEPE on ERK1/2 MAPK activation and the increase in BMP4 protein expression were nullified by pretreatment with increasing concentrations of p‐ERK1/2 inhibitor PD98059 in TM4 and primary Sertoli cells (Figures 6K and S8C).

Furthermore, the expression of proliferation‐ and differentiation‐related proteins, including PLZF, Lin28, c‐kit, and Stra8, was markedly inhibited by siRNA‐GPR120 compared with scrambled siRNA in the co‐culture and conditioned medium experiments (Figures 6L–O and S8D). Overall, these outcomes suggest that 12‐HEPE‐induced BMP4 expression in Sertoli cells contributes to spermatogonia proliferation and differentiation by activating the GPR120‐ERK1/2 signalling pathway in vitro.

### 3.7 Dietary 12‐HEPE restores spermatogenesis through the GPR120 pathway in vivo

**FIGURE 7:**
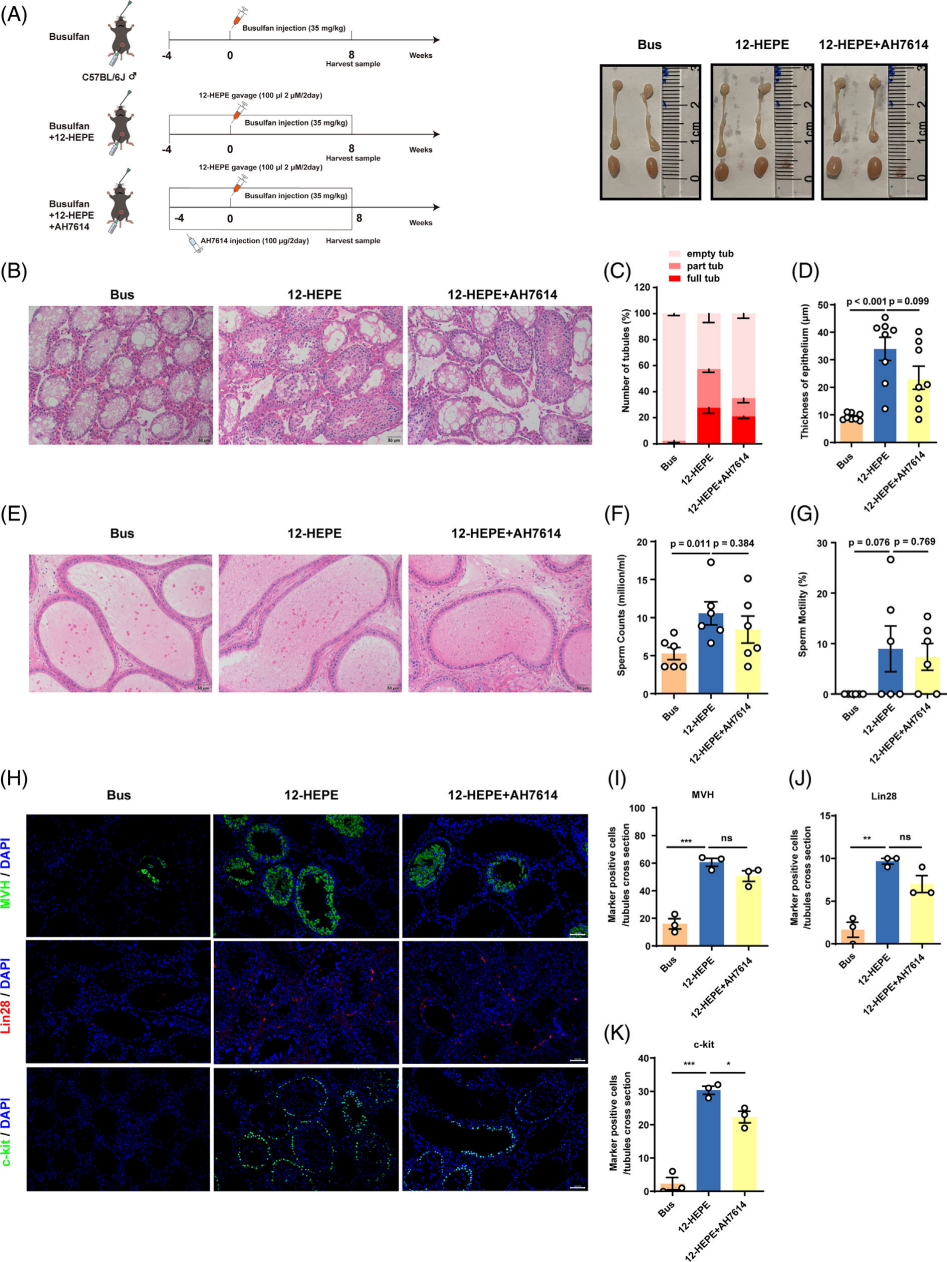
Dietary 12‐hydroxyeicosapentaenoic acid (12‐HEPE) restores spermatogenesis through the G protein‐coupled receptor 120 (GPR120) pathway. (A) Diagram of the animal experiments and images of testis morphology in all groups. (B) Representative hematoxylin and eosin (H&E) staining images of testicular cross‐sections from each group (*n* = 8; scale bar, 50 μm). Quantification of the quantity of seminiferous tubules exhibiting empty, partial or full spermatogenesis tubules (C) and tubule thickness (D) in relation to the control group. (E) Representative H&E staining images of caudal epididymis cross‐sections from each group (*n* = 8; scale bar, 50 μm). The sperm count (F) and motility (G) of model mice were assessed and compared with those of control mice. (H) Immunohistochemical staining indicated that MVH (green) was detected in newborn germ cells, Lin28 (red) was expressed in undifferentiated spermatogonia, and c‐kit (green) was found in differentiated spermatogonia in all groups (scale bar, 50 μm). (I–K) The count of MVH^+^, Lin28^+^, or c‐kit^+^ cells per cross‐section of tubules (*n* = 3). Compared with the control group: *p < 0.05; **p < 0.01; ***p < 0.001. The data are presented as the mean ± SEM.

To further determine the protective mechanisms of 12‐HEPE in vivo, busulfan‐treated male mice were given oral gavage of 12‐HEPE and intraperitoneal injection of AH7614. The experimental procedure was illustrated in the schematic diagram (Figure 7A). The weight ratios for the testis and epididymis of mice treated with AH7614 were similar to those of mice treated with 12‐HEPE (Figure S6). The serum sex hormone levels also showed no change between these two groups (Figure S7). Interestingly, in the AH7614‐treated group, tubules showing both partial and complete spermatogenesis exhibited a disrupted appearance (Figure 7B,C). Notably, the seminiferous tubule epithelium thickness was also reduced and was similar between AH7614‐treated mice and busulfan‐treated mice (Figure 7D). Additionally, the mice treated with GPR120 antagonist exhibited a decrease in both sperm count and motility when compared with the mice treated with 12‐HEPE (Figure 7E–G).

**FIGURE 8:**
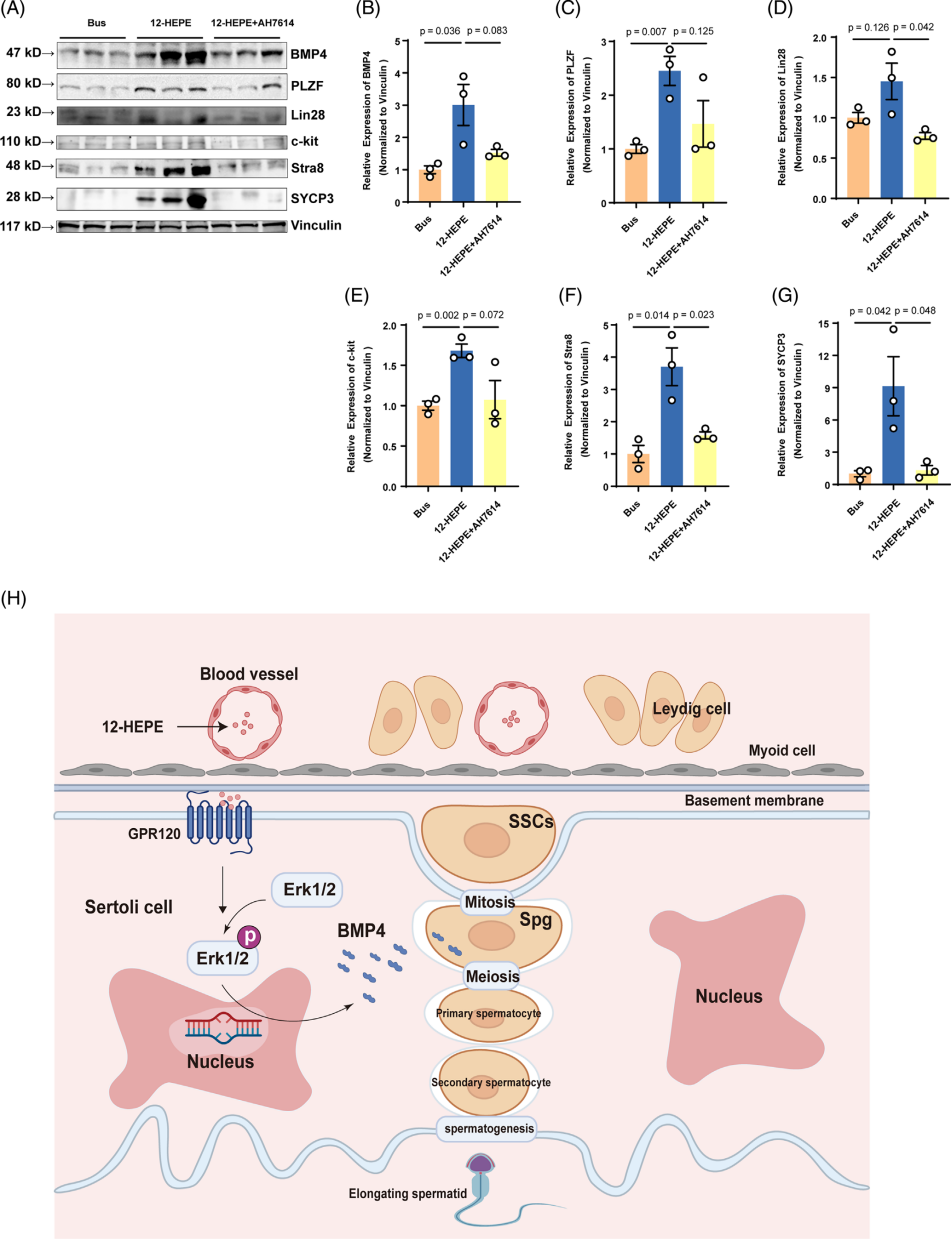
Dietary 12‐hydroxyeicosapentaenoic acid (12‐HEPE) restores spermatogonia proliferation and differentiation through the G protein‐coupled receptor 120 (GPR120) pathway. (A) The protein expression of bone morphogenic protein 4 (BMP4), PLZF, Lin28, c‐kit, Stra8, and SYCP3 in each group was assessed by western blotting (*n* = 3). (B–G) BMP4, PLZF, Lin28, c‐kit, Stra8, and SYCP3 protein expression was quantified by normalizing the levels of these proteins to the level of the internal control vinculin. The information is displayed as the mean ± SEM from a minimum of three separate trials. (H) Diagram illustrates how omega‐3 fatty acids and their metabolite 12‐HEPE restore spermatogenesis in busulfan‐treated mice by enhancing BMP4 secretion from Sertoli cells.

Furthermore, the numbers of germ cells (MVH), undifferentiated spermatogonia (Lin28), and differentiated spermatogonia (c‐kit) were lower in AH7614‐treated mice than in 12‐HEPE‐treated mice (Figure 7H–K). As compared to the 12‐HEPE‐treated group, the expression of the paracrine factor BMP4, undifferentiated spermatogonia‐related proteins (PLZF, Lin28), differentiated spermatogonia‐related proteins (c‐kit, Stra8), and a spermatocyte‐related protein (SYCP3) was inhibited in the GPR120 antagonist‐treated group (Figure 8A–G). Thus, these data indicate that 12‐HEPE‐stimulated endogenous spermatogenesis might involve the GPR120 pathway in vivo.

## 4 DISCUSSION

In this study, we observed that patients with severe dyszoospermia exhibited a decrease in omega‐3 levels in seminal plasma, suggesting that omega‐3 supplementation may restore spermatogenesis (Figure 1). In testis, Sertoli cells facilitate the conversion of EPA to DPA and DHA through undergoing a series of elongation and desaturation processes.^25^ The seminal plasma is the last stage of testicular metabolism, so the proportion of EPA content was lower, while the proportion of DHA and DPA content was higher (Figure 1B). Therefore, significant differences were presented by the levels of EPA metabolites, DHA, and DPA, between healthy individuals and sub‐fertile/infertile patients (Figure 1D,E). This study is the first to demonstrate that omega‐3 effectively protects spermatogonia from the effects of chemotherapy; specifically, it rescues spermatogonia function by enhancing BMP4 expression in Sertoli cells. We further found that some EPA metabolites, including 12‐HEPE, were produced at high levels in the testes of mice administered omega‐3 and that 12‐HEPE specifically induced BMP4 expression through the GPR120‐ERK1/2 signalling pathway in Sertoli cells (Figure 8H). Together, our data suggest that 12‐HEPE and GPR120, a receptor for long‐chain free fatty acids, could potentially serve as therapeutic targets for addressing spermatogenic dysfunction.

According to recent research, busulfan treatment can lead to the depletion of endogenous spermatogenic cells through various mechanisms, including DNA alkylation,^26^ disruption of vimentin filament distribution,^27^, ^28^ disruption of SSC differentiation,^29^ promotion of SSC dormancy,^30^ and induction of oxidative stress.^31^ Therefore, depleting endogenous germ cells via a single injection of busulfan has become the most commonly used technique for establishing animal models of disrupted spermatogenesis.^32^ Many in vivo studies have suggested that omega‐3 can alleviate testicular damage caused by environmental pollutants or metabolic disorders by alleviating oxidative stress,^33^ reducing apoptosis,^34^ repairing the blood‐testis barrier,^35^ and protecting against DNA damage.^36^ Notably, our results showed that omega‐3 could restore spermatogenesis by increasing the thickness of the spermatogenic tubule epithelium, alleviating spermatogenic cell apoptosis, and increasing the number of sperm in the caudal epididymis (Figure 2). Omega‐3 can enhance the differentiation of various types of cells, including skeletal muscle satellite cells,^37^ neural stem cells,^38^ adipocytes,^39^ and embryonic stem cells.^40^ The process of spermatogenesis involves successive phases of proliferation and differentiation, meiotic division, and spermatogenic stages.^41^ In mice, undifferentiated spermatogonia specifically express PLZF and Lin28, as reported in studies.^42^, ^43^, ^44^ C‐kit and Stra8 are considered markers of differentiated spermatogonia.^45^, ^46^ SYCP3, a protein complex found in synapses, has a crucial function in the process of meiosis and is specifically present in the initial stages of spermatocytes.^47^, ^48^ MVH (DDX4) is characteristically present in cells that are part of the germ cell lineage.^49^ The aforementioned proteins were utilized to identify the particular phase of spermatogenesis from which these germ cells originated. According to our additional discoveries, omega‐3‐activated endogenous spermatogonia self‐renewal and differentiation after busulfan treatment by promoting an increase in the numbers of undifferentiated spermatogonia (PLZF and Lin28) and differentiated spermatogonia (c‐kit and Stra8; Figure 3), implying a role for omega‐3 in the spermatogenesis process following injury to the male reproductive system. In addition, our data indicated that omega‐3 markedly increased the protein expression of the spermatocyte marker SYCP3. One can speculate that omega‐3 may contribute to the function of spermatocytes, but this hypothesis needs additional investigation in the coming times.

Oxidative stress is a key factor in infertility of patients with severe dyszoospermia.^50^ Indeed, we confirmed that busulfan stimulated ROS production in spermatogonia but not in Sertoli cells during an in vitro study, indicating that spermatogonia were more vulnerable to oxidative stress damage and suppression of proliferation and differentiation. Spermatogonia maintain a close connection with Sertoli cells, which offer both structural assistance and paracrine substances to self‐renewal and differentiation of spermatogonia. During the early postnatal period, Sertoli cells secrete the paracrine factor BMP4, which stimulates undifferentiated spermatogonia to undergo both mitosis and differentiation, inducing Kit expression.^6^ On the other hand, retinoic acid (RA) signalling can touch off the differentiation of spermatogonia and drive meiotic origination, which is regulated by FSH signalling in Sertoli cells within the testis.^51^ The oxidation of vitamin A to retinol by retinol dehydrogenase 10 (Rdh10) in Sertoli cells is critical for RA biosynthesis.^52^ Nevertheless, it is unclear whether the omega‐3‐mediated recovery of spermatogenesis was due to other specific factors in addition to a reduction in oxidative stress. Thus, we further investigated which omega‐3 metabolites showed an increase in concentration after busulfan treatment and specifically induced the production of paracrine factors and RA signalling in Sertoli cells. PUFAs undergo catalysis to produce different metabolites via the cytochrome P450, LOX, and cyclooxygenase (COX) pathways.^53^, ^54^ It has become increasingly evident that a large part of the physiological functions of ARA, EPA, and DHA are mediated via their oxylipins, which regulates numerous physiological processes, including inflammation, pain, and blood coagulation.^55^, ^56^ Our metabonomic data revealed that dietary omega‐3 elevated the levels of the anti‐inflammatory PUFA EPA and its metabolites but decreased the levels of the proinflammatory PUFA ARA and its metabolites in testicular tissue (Figure 4). Our findings demonstrate that the increase in 12‐HEPE levels induced by the metabolites of EPA contributes to an increase in Sertoli cell‐derived expression of BMP4 but not Rhd10, leading to spermatogonia proliferation and differentiation in vitro (Figure 5).

Although we demonstrated that the testes do not produce 12‐HEPE, it is plausible that additional cellular including adipocytes, platelets, or myeloid cells, could also affect the presence of 12‐LOX metabolites in the bloodstream due to the high expression of 12‐LOX in these specific cell types.^57^, ^58^, ^59^ These results suggest that 12‐HEPE in the testes might originate from the circulatory system. Substantial studies recently have shown that GPR120 is a functional receptor for omega‐3 PUFAs involved in regulating metabolic, endocrine, and immune functions.^60^, ^61^, ^62^, ^63^ The recognition of various double‐bond positions of omega‐3 PUFAs and the connection of ligand recognition to distinct effector coupling were attributed to aromatic residues located within the GPR120 ligand pocket.^64^ In our research, we noticed that omega‐3 group exhibited a heightened GPR120 expression in testicular Sertoli cells compared with the control group (Figure 6A), implying that the function of Sertoli cells could be regulated by the binding of 12‐HEPE to GPR120. In addition, our work demonstrated that GPR120 was also located in Leydig cells, suggesting that 12‐HEPE might also thought to help Leydig cells function. It was previously reported that the activation of GPR120/ ERK pathway was found to enhance testosterone production through LA stimulation in Leydig cells.^65^ However, we found no change with testosterone levels in omega‐3‐treated mice. It was previously reported that Consistent with a previous study that demonstrated that 12‐HEPE can reinforce GPR activity and ultimately increase glucose uptake by brown adipocytes,^66^ we confirmed that 12‐HEPE promoted BMP4 expression via GPR120 activation in Sertoli cells (Figure 6). It is not clear from our study whether 12‐HEPE might improve nutritional support to germ cells by enhancing glucose uptake and ultimately lactate secretion from Sertoli cells. Earlier studies indicated that PUFAs and their metabolites significantly increased GPR activity and then activated ERK MAPK pathway in round spermatids, intestinal epithelial cells, and umbilical vein endothelial cells.^67^, ^68^, ^69^ Recent research has shown that the ERK1/2 pathway regulates the proliferation, maturation, and paracrine function of Sertoli cells.^70^, ^71^ In agreement, our current investigation revealed that 12‐HEPE accelerates BMP4 expression through excitation of the GPR120‐ERK1/2 pathway. western blot analysis revealed a notable increase in the expression of p‐ERK after 60 min of treatment with 12‐HEPE, which was sustained at 90 min. Both siRNA‐GPR120 and AH7614, a selective GPR120 antagonist, significantly inhibited ERK activation induced by 12‐HEPE in Sertoli cells. In addition, ERK1/2 activation was completely inhibited by PD98059. This ERK inhibitor also blocked the subsequent induction of BMP4 expression (Figure 6). Notably, co‐administration of 12‐HEPE and AH7614 hindered the capacity of 12‐HEPE to restore the presence of the testicular paracrine factor BMP4 and endogenous spermatogonia self‐renewal and differentiation in mice treated with busulfan (Figures 7 and 8). Hence, we conclude that the safeguarding properties of 12‐HEPE on sperm production are partly facilitated by its interaction with GPR120. Several recent studies have suggested that 15‐HEPE can attenuate the production of inflammatory markers through peroxisome proliferator‐activated receptor gamma (PPARγ).^72^, ^73^ We speculate that PPARγ might also be a potential target of 12‐HEPE.

Although we studied the regulatory effect of the lipid metabolites 12‐HEPE, derived from EPA, on the paracrine function of Sertoli cells, there still are more areas we can explore. First, it is that the contributions of two other metabolites, namely, 15‐HEPE and 5,6‐DiHETE, to nutrition metabolism and the blood‐testis barrier remain to be determined. Second, although we studied normal spermatozoa obtained from mice that received dietary omega‐3 supplementation or 12‐HEPE busulfan treatment, the fertility and condition of healthy offspring, including physiological parameters, basic metabolism, and life expectancy, need to be assessed to evaluate the long‐term effects of omega‐3 and 12‐HEPE on Sertoli and germline cells in the future. Finally, BMP4 is crucial for establishing the germ cell lineage before birth, and probably in early spermatogenesis. It is necessary to discuss the role of BMP4 in steady‐state spermatogenesis. It is important to note that BMP4 was reportedly detected in human Sertoli cells obtained from the testicular tissue of NOA patients and that BMP4 transcription and protein expression levels were considerably reduced in Sertoli cells from NOA patients compared with healthy individuals.^74^, ^75^ The alteration of BMP4 expression in testis Sertoli cells may have an impact on the process of spermatogenesis. However, considering the further damage to testicular function caused by testicular biopsy, we thought that metabolic detection technology may be able to better predict spermatogenesis potential through non‐invasive detection of seminal plasma metabolites. We assessed the reduction in the levels of seminal plasma omega‐3 PUFAs in patients with severe dyszoospermia in our study. Further investigation is required to assess the value of PUFA metabolites, including 12‐HEPE, in assessing testicular spermatogenesis function.

For our research, we chosen oral omega‐3 PUFAs and 12‐HEPE supplementation for the experiments, but the perspective to use alternative formulations and other administration routes of nanomedicines can be indicated in the context of evaluating the efficacy of different drug delivery strategies. This could involve developing supramolecular assemblies that incorporate PUFA‐chain plasmalogens into nanostructured mixtures with lyotropic lipids.^76^ Another perspective for the continuation of this work would be to combine omega‐3 PUFAs with an antioxidant such as curcumin. This combination of drugs has been shown to enhance the expression of neurotrophic factors.^77^ It may be expected that dual drug formulations (12‐HEPE and antioxidant) may be beneficial for the outcome of spermatogonia proliferation and differentiation at much lower doses of administered omega‐3 PUFAs.

## 5 CONCLUSION

Our study demonstrates that continuous supplementation with omega‐3 can effectively accelerate the recovery of spermatogenesis following chemotherapy by enhancing the self‐renewal and differentiation of the remaining spermatogonia. We reveal that the endocrine factor 12‐HEPE facilitates the proliferation and differentiation of spermatogonia via activating the GPR120‐ERK1/2‐BMP4 pathway in Sertoli cells. Given the harmless characteristics of 12‐HEPE, along with its observed metabolic impacts and the mechanisms of action revealed in our investigation, 12‐HEPE or 12‐HEPE‐mimetics showed a significant promise in safeguarding the reproductive abilities of boy individuals diagnosed with cancer. In combination, our data not only uncover an unidentified mechanism involving PUFA metabolites and the spermatogenesis microenvironment, but also offer a safe and effective novel drug delivery strategy for individuals with spermatogenic dysfunction.

## AUTHOR CONTRIBUTIONS

*Conceptualization*: Bing Yao, Chaojun Li, and Jun Jing. Methodology: Jun Jing, Lei Ouyang, Hong Zhang, Rujun Ma, and Xie Ge. *Investigation*: Jun Jing, Lei Ouyang, Hong Zhang, Kuan Liang, Ting Tang, Tongmin Xue, Jiaming Shen, Zhou Li, Lu Zheng, Zhang Qian, and Shanshan Sun. *Data analysis*: Jun Jing, Lei Ouyang, and Hong Zhang. *Visualization*: Jun Jing, Lei Ouyang, Ting Tang, and Shanmeizi Zhao. *Clinical expertise provision*: Jun Jing, Jinzhao Ma, Wei Zhao, and Yifeng Ge. *Experimental technical support*: Jiaming Shen, Rujun Ma, Xie Ge, Jinzhao Ma, Yang Yang, Wei Zhao, Jing Wu, and Yifeng Ge. *Funding acquisition*: Bing Yao, Chaojun Li, Li Chen, and Jun Jing. *Project administration*: Jun Jing and Lei Ouyang. *Supervision*: Bing Yao, Chaojun Li, and Li Chen. *Writing—original draft*: Jun Jing and Lei Ouyang. *Writing—review and editing*: Jun Jing, Lei Ouyang, Hong Zhang, Jing Wu, Jinzhao Ma, and Shanmeizi Zhao. *Revising*: Jun Jing, Lei Ouyang, Hong Zhang, Jiaming Shen, and Zhou Li. All authors read and approved the final article.

## FUNDING INFORMATION

This work was supported by the National Key Research and Development Program of China (grant numbers 2018YFC1004700 and 2018YFC1003800), the National Natural Science Foundation of China (grant numbers U22A20277, 81701431, 82271687, 81901547, 82001618, 82001535, 81973965, and 81971373), Postgraduate Research & Practice Innovation Program of Jiangsu Province (grant number KYCX22_1826), Independent Research Project of State Key Laboratory of Reproductive Medicine (grant number SKLRM‐2022D3), and Jiangsu Provincial Medical Key Discipline Cultivation Unit (grant number JSDW202215).

## CONFLICT OF INTEREST STATEMENT

The authors declare that they have no competing interests.

## Supporting information


**FIGURE S1.** Effects of dietary omega‐3 supplementation on the reproductive organ/body weight ratio of mice with busulban administered. The testicular weight (A), testicular organ index (B), epididymis weight (C) and epididymis organ index (D) of model mice were measured (*n* = 6 per group) and compared with those of control mice. The data are presented as the mean ± SEM.

**FIGURE S2.** Effect of dietary omega‐3 on serum sex hormone levels in mice. Serum testosterone (A), serum inhibin B (B), serum LH (C), and serum FSH (D) levels were measured by ELISA (*n* = 6 per group) and compared with those in control mice. The data are presented as the mean ± SEM.

**FIGURE S3.** Dietary omega‐3 reduces lipid metabolite levels in testicular tissue. (A–I) The metabolites of ARA, the levels of 5‐HETE, 9‐HETE, 15‐HETE, 16‐HETE, 5‐HETrE, 5‐iso PGF2VI, 5s,15s‐DiHETE, 15‐oxoETE, and LTB4 in testicular tissue from the omega‐3‐treated group were significantly lower than those in testicular tissue from the busulfan‐treated groups (*n* = 6 per group). The data are presented as the mean ± SEM.

**FIGURE S4.** Busulban increases ROS production and inhibits the proliferation and differentiation of spermatogonia. (A) ROS levels in TM4 and GC‐1 cells were analysed after treatment with busulfan using IF (scale bar, 20 μm). (B) GC‐1 cells were treated with busulfan or H_2_O_2_ for 24 h, and the mRNA expression of PLZF, Lin28, c‐kit, Stra8, Rdh10, Aldh1a2, and CRABP1 was examined by qRT‐PCR. Compared with the control group: *p < 0.05; **p < 0.01. (C) The proliferation of busulfan‐treated GC‐1 cells was analysed by the EdU incorporation assay (scale bar, 50 μm). (D) The number of EdU+ cells in each group. Compared with the control group: ***p < 0.001.

**FIGURE S5.** Effects of 12‐HEPE on the proliferation and differentiation of spermatogonia. GC‐1 cells were treated with 10^−4^ M busulfan with or without 12‐HEPE for 24 h, and then the protein expression of PLZF, Lin28, c‐kit, and Stra8 was measured by western blotting and compared with that in group treated with busulfan alone.

**FIGURE S6.** Effects of dietary 12‐HEPE on the reproductive organ/body weight ratio of mice with busulban administered. The testicular weight (A), testicular organ index (B), epididymis weight (C) and epididymis organ index (D) of model mice were measured (*n* = 6 per group) and compared with those of busulfan‐treated mice. The data are presented as the mean ± SEM.

**FIGURE S7.** Effect of dietary 12‐HEPE on serum sex hormone levels in mice with busulban administered. Serum testosterone (A), serum inhibin B (B), serum LH (C) and serum FSH (D) levels were measured by ELISA (*n* = 6 per group) and compared with those in busulfan‐treated mice. The data are presented as the mean ± SEM.

**FIGURE S8.** 12‐HEPE up‐regulates BMP4 via GPR120‐ERK in primary Sertoli cells and protects primary spermatogonia proliferation and differentiation. (A) Schematic of the co‐culture system including primary spermatogonia and Sertoli cells treated with busulfan or Bus+12‐HEPE. The protein expression of germ cell markers was quantified by western blotting in primary spermatogonia. (B) The primary Sertoli cells were transfected with siRNA‐GPR120‐3 or scrambled siRNA and then treated with or without 12‐HEPE for 24 h. GPR120, BMP4 and the levels of p‐ERK1/2 and t‐ERK1/2 was assessed by western blotting. (C) The primary Sertoli cells were pretreated with various concentrations of PD98059 (a p‐ERK inhibitor) for 2 h and then treated with 12‐HEPE for another 24 h. Then, BMP4 protein expression was evaluated by western blotting. (D) Schematic of the co‐culture system including primary spermatogonia and Sertoli cells transfected with siRNA‐GPR120‐3 or scrambled siRNA and then treated with busulfan and 12‐HEPE for 24 h. The protein expression of germ cell markers was evaluated by western blotting in primary spermatogonia.

**TABLE S1.** Summary of the clinical parameters of all study participants.
**TABLE S2.** siRNA sequences.
**TABLE S3.** Primer sequences for qPCR.

## Data Availability

All data generated or analysed during this study are available from the corresponding author upon reasonable request.
