# Supplementary figures and images for: Omega‐3 polyunsaturated fatty acids and its metabolite 12‐HEPE rescue busulfan disrupted spermatogenesis via target to GPR120
Source: Cell Prolif. 2023 Sep 24;57(2):e13551. doi: 10.1111/cpr.13551 (PMC10849791; doi:10.1111/cpr.13551)

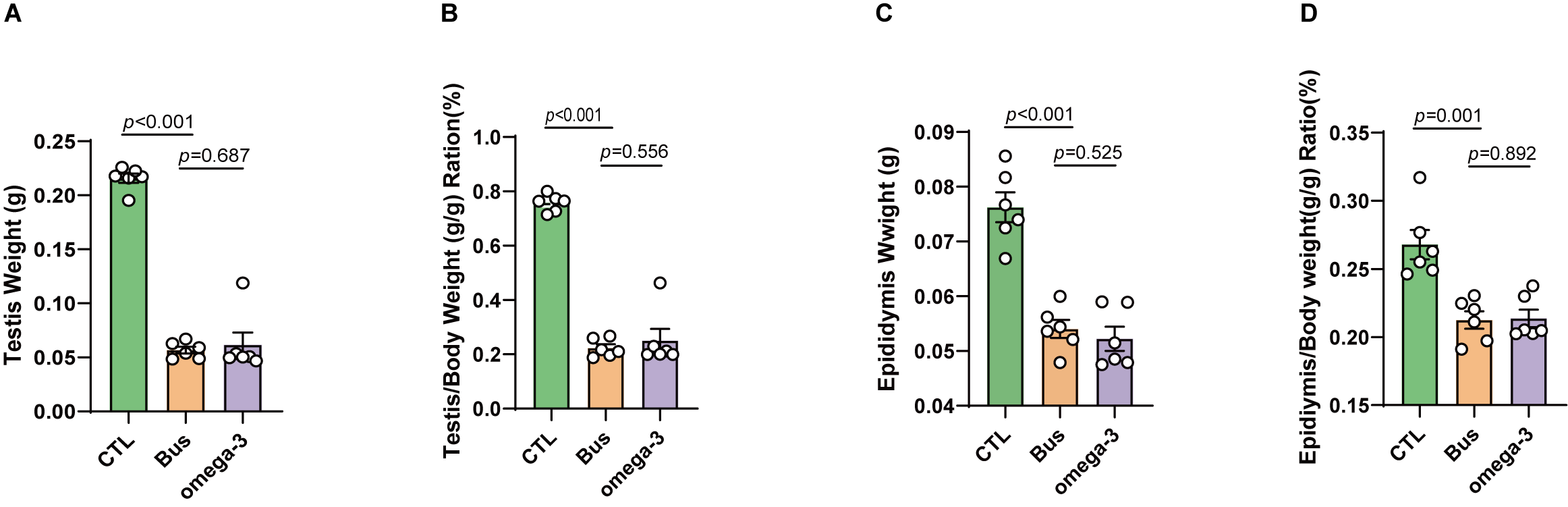

Supplement: Supplementary file 1 — FIGURE S1. Effects of dietary omega‐3 supplementation on the reproductive organ/body weight ratio of mice with busulban administered. The testicular weight (A), testicular organ index (B), epididymis weight (C) and epididymis organ index (D) of model mice were measured (n = 6 per group) and compared with those of control mice. The data are presented as the mean ± SEM. [file CPR-57-e13551-s004.tif]

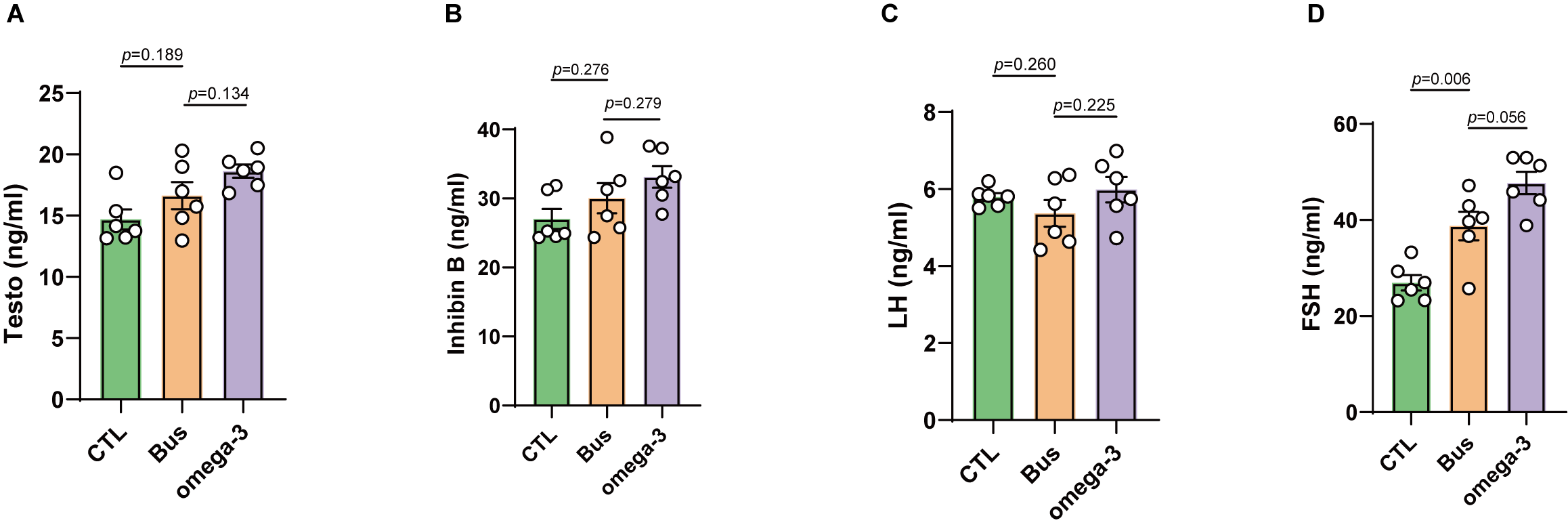

Supplement: Supplementary file 2 — FIGURE S2. Effect of dietary omega‐3 on serum sex hormone levels in mice. Serum testosterone (A), serum inhibin B (B), serum LH (C), and serum FSH (D) levels were measured by ELISA (n = 6 per group) and compared with those in control mice. The data are presented as the mean ± SEM. [file CPR-57-e13551-s001.tif]

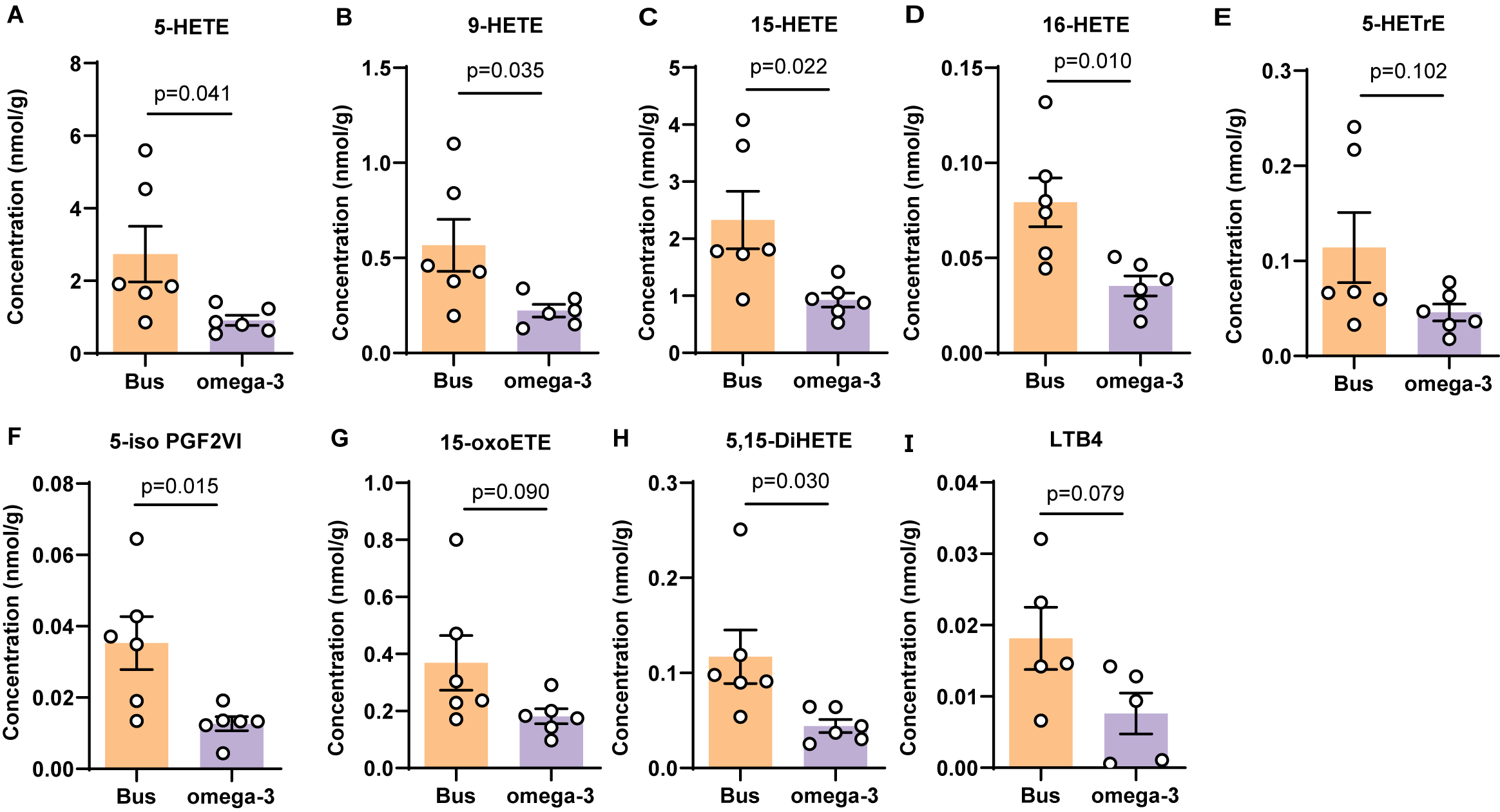

Supplement: Supplementary file 3 — FIGURE S3. Dietary omega‐3 reduces lipid metabolite levels in testicular tissue. (A–I) The metabolites of ARA, the levels of 5‐HETE, 9‐HETE, 15‐HETE, 16‐HETE, 5‐HETrE, 5‐iso PGF2VI, 5s,15s‐DiHETE, 15‐oxoETE, and LTB4 in testicular tissue from the omega‐3‐treated group were significantly lower than those in testicular tissue from the busulfan‐treated groups (n = 6 per group). The data are presented as the mean ± SEM. [file CPR-57-e13551-s008.tif]

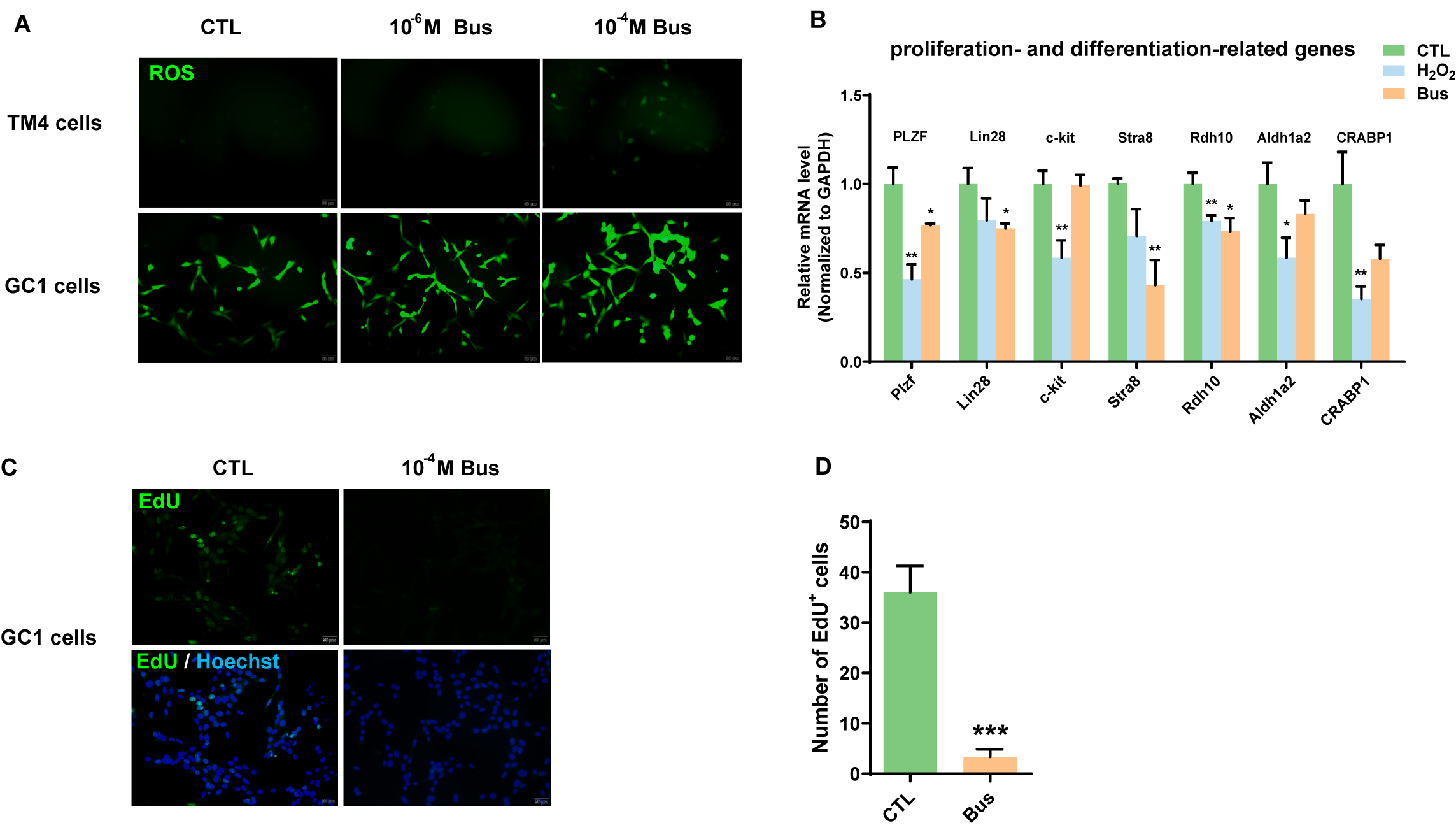

Supplement: Supplementary file 4 — FIGURE S4. Busulban increases ROS production and inhibits the proliferation and differentiation of spermatogonia. (A) ROS levels in TM4 and GC‐1 cells were analysed after treatment with busulfan using IF (scale bar, 20 μm). (B) GC‐1 cells were treated with busulfan or H2O2 for 24 h, and the mRNA expression of PLZF, Lin28, c‐kit, Stra8, Rdh10, Aldh1a2, and CRABP1 was examined by qRT‐PCR. Compared with the control group: *p < 0.05; **p < 0.01. (C) The proliferation of busulfan‐treated GC‐1 cells was analysed by the EdU incorporation assay (scale bar, 50 μm). (D) The number of EdU+ cells in each group. Compared with the control group: ***p < 0.001. [file CPR-57-e13551-s006.tif]

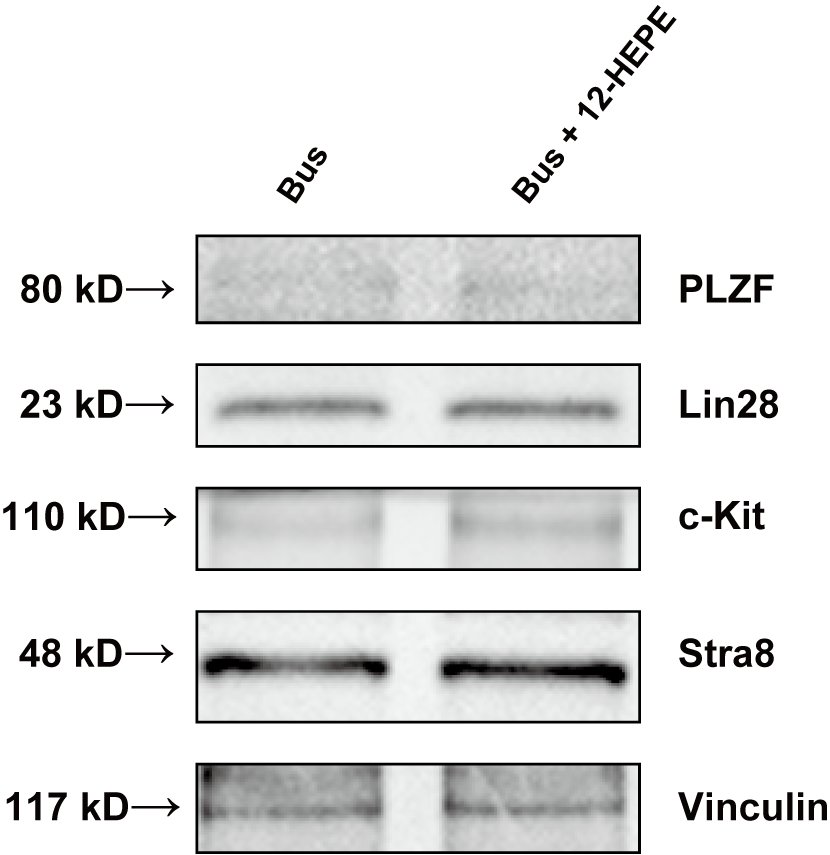

Supplement: Supplementary file 5 — FIGURE S5. Effects of 12‐HEPE on the proliferation and differentiation of spermatogonia. GC‐1 cells were treated with 10−4 M busulfan with or without 12‐HEPE for 24 h, and then the protein expression of PLZF, Lin28, c‐kit, and Stra8 was measured by western blotting and compared with that in group treated with busulfan alone. [file CPR-57-e13551-s002.tif]

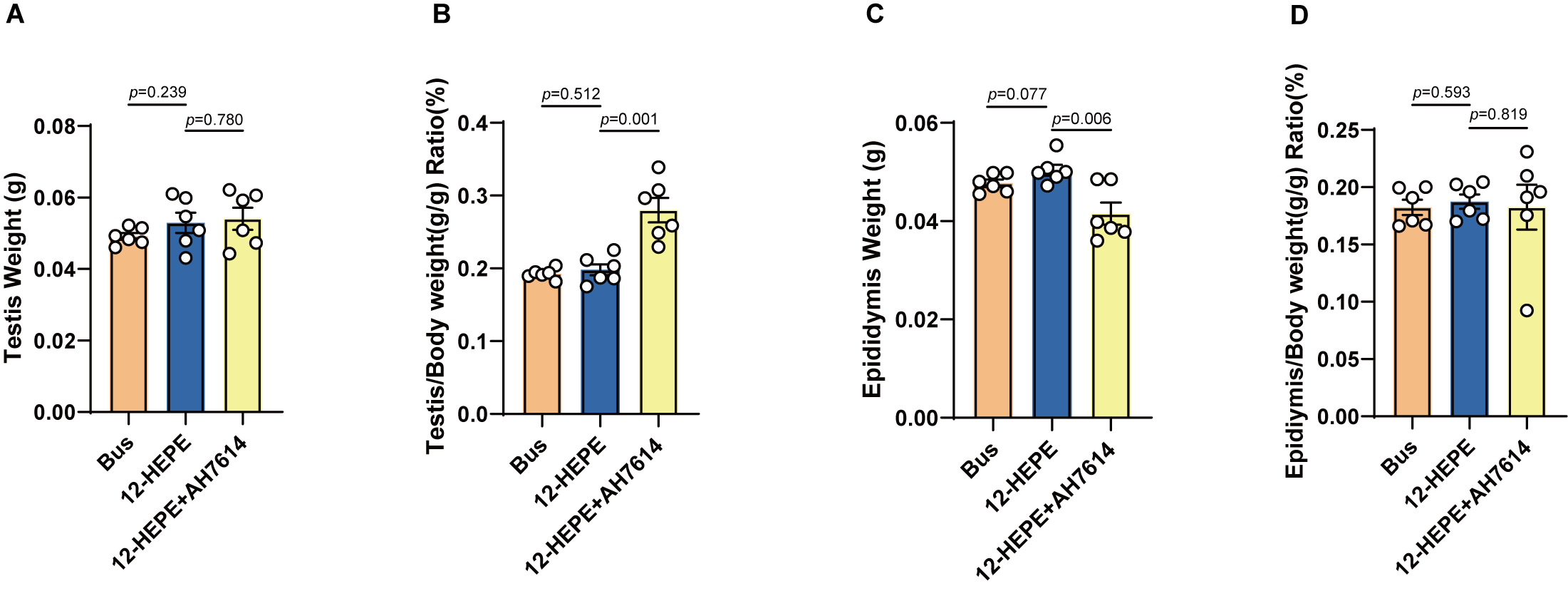

Supplement: Supplementary file 6 — FIGURE S6. Effects of dietary 12‐HEPE on the reproductive organ/body weight ratio of mice with busulban administered. The testicular weight (A), testicular organ index (B), epididymis weight (C) and epididymis organ index (D) of model mice were measured (n = 6 per group) and compared with those of busulfan‐treated mice. The data are presented as the mean ± SEM. [file CPR-57-e13551-s005.tif]

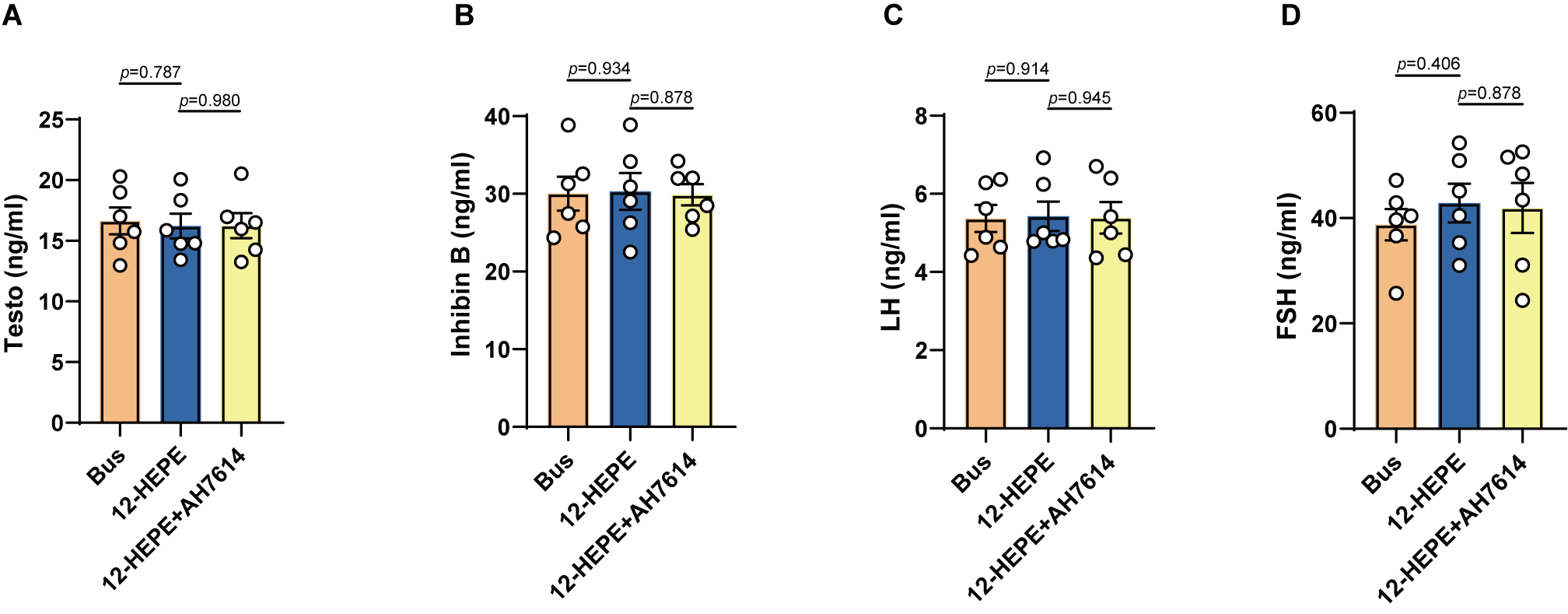

Supplement: Supplementary file 7 — FIGURE S7. Effect of dietary 12‐HEPE on serum sex hormone levels in mice with busulban administered. Serum testosterone (A), serum inhibin B (B), serum LH (C) and serum FSH (D) levels were measured by ELISA (n = 6 per group) and compared with those in busulfan‐treated mice. The data are presented as the mean ± SEM. [file CPR-57-e13551-s009.tif]

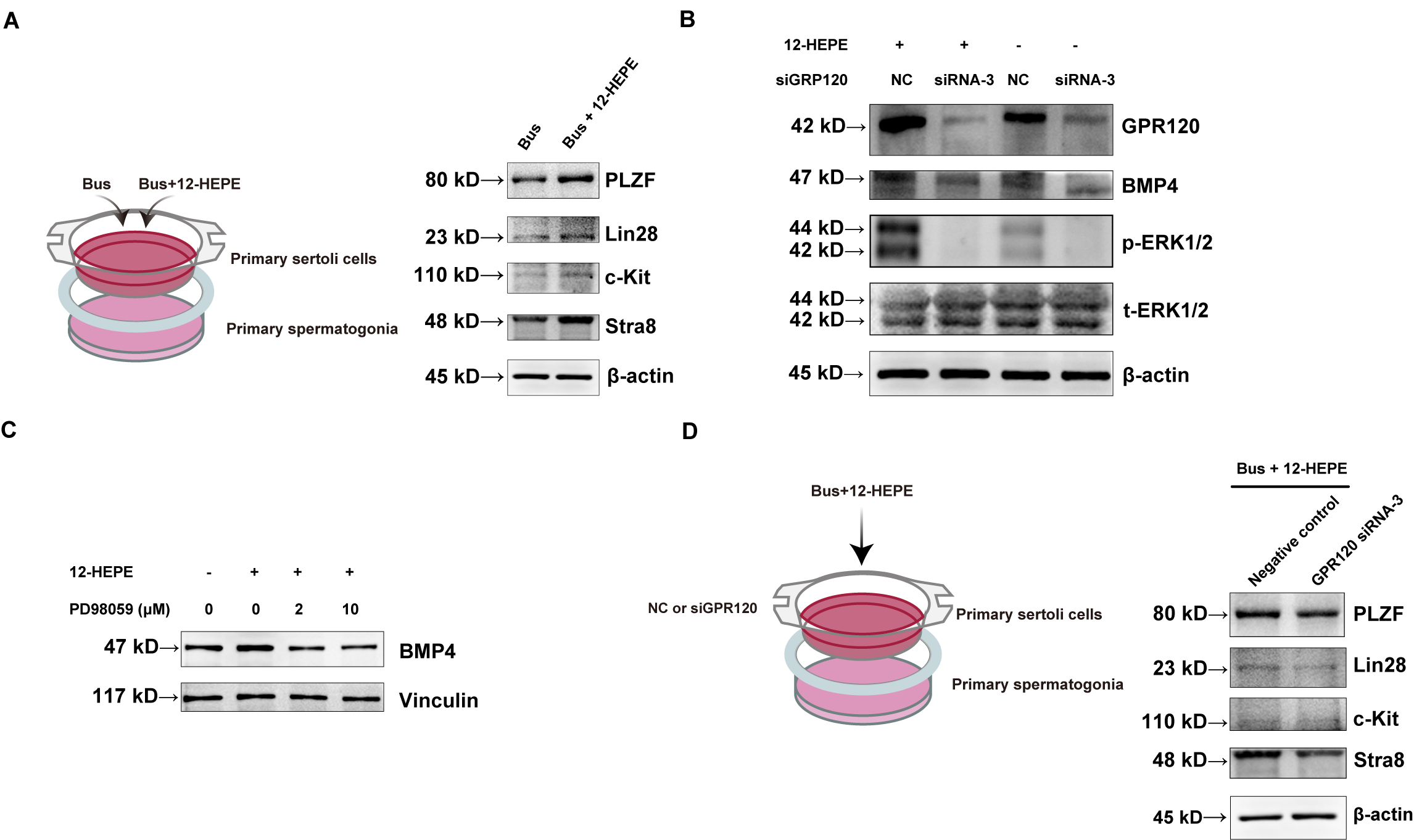

Supplement: Supplementary file 8 — FIGURE S8. 12‐HEPE up‐regulates BMP4 via GPR120‐ERK in primary Sertoli cells and protects primary spermatogonia proliferation and differentiation. (A) Schematic of the co‐culture system including primary spermatogonia and Sertoli cells treated with busulfan or Bus+12‐HEPE. The protein expression of germ cell markers was quantified by western blotting in primary spermatogonia. (B) The primary Sertoli cells were transfected with siRNA‐GPR120‐3 or scrambled siRNA and then treated with or without 12‐HEPE for 24 h. GPR120, BMP4 and the levels of p‐ERK1/2 and t‐ERK1/2 was assessed by western blotting. (C) The primary Sertoli cells were pretreated with various concentrations of PD98059 (a p‐ERK inhibitor) for 2 h and then treated with 12‐HEPE for another 24 h. Then, BMP4 protein expression was evaluated by western blotting. (D) Schematic of the co‐culture system including primary spermatogonia and Sertoli cells transfected with siRNA‐GPR120‐3 or scrambled siRNA and then treated with busulfan and 12‐HEPE for 24 h. The protein expression of germ cell markers was evaluated by western blotting in primary spermatogonia. [file CPR-57-e13551-s003.tif]
