# Supplementary material for: Omega‐3 polyunsaturated fatty acids and its metabolite 12‐HEPE rescue busulfan disrupted spermatogenesis via target to GPR120
Source: Cell Prolif. 2023 Sep 24;57(2):e13551. doi: 10.1111/cpr.13551 (PMC10849791; doi:10.1111/cpr.13551)
Supplement: Supplementary file 9 — TABLE S1. Summary of the clinical parameters of all study participants. TABLE S2. siRNA sequences. TABLE S3. Primer sequences for qPCR. [file CPR-57-e13551-s007.docx]

**Table S1. Summary of the clinical parameters of all study participants.**

| **No.** | **Age** | **Sperm concentration (10^6/mL)** | **Total motility (%)** | **Progressive motility (%)** | **Normal sperm percentage (%)** | **Inhibin B (pg/mL)** | **Type** |
| --- | --- | --- | --- | --- | --- | --- | --- |
| 1 | 23 | 99.75 | 49.66 | 32.55 | 7.84 | 180.00 | Normal |
| 2 | 35 | 51.88 | 46.77 | 34.52 | 6.93 | 142.00 | Normal |
| 3 | 28 | 67.95 | 57.64 | 45.32 | 9.39 | 129.00 | Normal |
| 4 | 28 | 148.79 | 65.35 | 40.27 | 4.33 | 130.00 | Normal |
| 5 | 33 | 30.58 | 44.16 | 37.96 | 5.88 | 103.00 | Normal |
| 6 | 31 | 71.31 | 59.62 | 44.60 | 5.21 | 113.00 | Normal |
| 7 | 33 | 26.00 | 42.49 | 39.48 | 4.05 | 133.00 | Normal |
| 8 | 29 | 22.02 | 41.50 | 34.66 | 7.21 | 97.80 | Normal |
| 9 | 32 | 80.83 | 40.37 | 35.85 | 4.25 | 196.00 | Normal |
| 10 | 31 | 69.63 | 41.06 | 34.13 | 4.46 | 127.00 | Normal |
| 11 | 29 | 105.61 | 41.68 | 32.65 | 7.04 | 117.00 | Normal |
| 12 | 32 | 60.08 | 40.95 | 34.82 | 5.94 | 209.00 | Normal |
| 13 | 27 | 79.83 | 66.67 | 54.09 | 4.90 | 178.00 | Normal |
| 14 | 32 | 43.97 | 49.49 | 42.39 | 6.37 | 137.00 | Normal |
| 15 | 28 | 28.01 | 40.84 | 33.86 | 5.91 | 104.00 | Normal |
| 16 | 31 | 81.35 | 48.77 | 37.65 | 4.43 | 145.00 | Normal |
| 17 | 29 | 45.53 | 54.78 | 44.12 | 6.97 | 94.20 | Normal |
| 18 | 26 | 94.74 | 54.06 | 42.40 | 5.45 | 113.00 | Normal |
| 19 | 31 | 39.16 | 49.57 | 43.59 | 6.34 | 98.50 | Normal |
| 20 | 32 | 94.06 | 40.57 | 35.94 | 4.43 | 162.00 | Normal |
| 21 | 29 | 44.85 | 60.07 | 54.48 | 6.40 | 93.30 | Normal |
| 22 | 29 | 35.15 | 53.81 | 48.57 | 4.95 | 189.00 | Normal |
| 23 | 29 | 46.70 | 61.65 | 51.25 | 5.66 | 153.00 | Normal |
| 24 | 30 | 40.09 | 44.04 | 35.07 | 5.58 | 175.00 | Normal |
| 25 | 32 | 60.76 | 48.21 | 42.15 | 5.58 | 115.00 | Normal |
| 26 | 32 | 0 | － | － | － | 5.38 | NOA |
| 27 | 26 | 0 | － | － | － | 10.31 | NOA |
| 28 | 28 | 0 | － | － | － | 13.30 | NOA |
| 29 | 25 | 0 | － | － | － | 11.20 | NOA |
| 30 | 31 | 0 | － | － | － | 11.50 | NOA |
| 31 | 20 | 0 | － | － | － | 12.00 | NOA |
| 32 | 22 | 0 | － | － | － | 12.20 | NOA |
| 33 | 33 | 0 | － | － | － | 12.80 | NOA |
| 34 | 27 | 0 | － | － | － | 12.30 | NOA |
| 35 | 28 | 0 | － | － | － | 12.50 | NOA |
| 36 | 25 | 0 | － | － | － | 12.90 | NOA |
| 37 | 33 | 0 | － | － | － | 14.10 | NOA |
| 38 | 34 | 0 | － | － | － | 15.50 | NOA |
| 39 | 30 | 0 | － | － | － | 16.10 | NOA |
| 40 | 31 | 0 | － | － | － | 16.70 | NOA |
| 41 | 27 | 0.33 | － | － | － | 18.79 | EO |
| 42 | 28 | 0 | － | － | － | 19.90 | NOA |
| 43 | 29 | 0.11 | － | － | － | 20.50 | EO |
| 44 | 25 | 0 | － | － | － | 21.40 | NOA |
| 45 | 24 | 0 | － | － | － | 21.40 | NOA |
| 46 | 34 | 0 | － | － | － | 22.40 | NOA |
| 47 | 43 | 0 | － | － | － | 29.10 | NOA |
| 48 | 30 | 0.50 | － | － | － | 30.40 | EO |
| 49 | 25 | 0.33 | － | － | － | 39.20 | EO |
| 50 | 26 | 0 | － | － | － | 52.60 | NOA |

**Table S2. siRNA sequences.**

|  | siRNA sequence for CD36 (5’ to 3’) |
| --- | --- |
| GPR120 siRNA1 | CGAAAUGACUUGUCUGUUATT  UAACAGACAAGUCAUUUCGTT |
| GPR120 siRNA2 | ACGAAAGCAUCGCGGAAGATT  UCUUCCGCGAUGCUUUCGUTT |
| GPR120 siRNA3 | GGGCAACGUGUGUGCUCUATT  UAGAGCACACACGUUGCCCTT |

**Table S3. Primer sequences for qPCR.**

| Mouse primer sequences | |
| --- | --- |
| **Gene** | Sequence (5’ to 3’) |
| GPR120 | Forward Primer: ACCAAGTCAATCGCACCCAC  Reverse Primer: GTGAGACGACAAAGATGAGCC |
| Zbtb16 | Forward Primer: CTGGGACTTTGTGCGATGTG  Reverse Primer: TCCTTCAGTCTGAGGTCGTTG |
| Lin28B | Forward Primer: TAGGTGGAGACGGCAGGATTT  Reverse Primer: ACCACAGTTGTAGCATCTTGGA |
| Stra8 | Forward Primer: ACAACCTAAGGAAGGCAGTTTAC  Reverse Primer: GACCTCCTCTAAGCTGTTGGG |
| Kit | Forward Primer: GCCTGACGTGCATTGATCC  Reverse Primer: AGTGGCCTCGGCTTTTTCC |
| Gdnf | Forward Primer: TCCAACTGGGGGTCTACGG |
|  | Reverse Primer: GCCACGACATCCCATAACTTCAT |
| BMP4 | Forward Primer: TTCCTGGTAACCGAATGCTGA |
| Nrg3  KitL  Rdh10  Aldh1a1  Aldh1a2  Crabp1  Crabp2  GAPDH | Reverse Primer: CCTGAATCTCGGCGACTTTTT  Forward Primer: TTACGCTGTAGCGACTGCATC  Reverse Primer: GCCTACCACGATCCATTTAAGC  Forward Primer: GAATCTCCGAAGAGGCCAGAA  Reverse Primer: GCTGCAACAGGGGGTAACAT  Forward Primer: GAACATCGTAGTGGAGTTCTTCG  Reverse Primer: CGGTCTCCTCATTGCTCTGC  Forward Primer: ATACTTGTCGGATTTAGGAGGCT  Reverse Primer: GGGCCTATCTTCCAAATGAACA  Forward Primer: CAGAGAGTGGGAGAGTGTTCC  Reverse Primer: CACACAGAACCAAGAGAGAAGG    Forward Primer: CAGCAGCGAGAATTTCGACGA  Reverse Primer: CGCACAGTAGTGGATGTCTTGA  Forward Primer: ATGCCTAACTTTTCTGGCAACT  Reverse Primer: GCACAGTGGTGGAGGTTTTGA  Forward Primer: AGGTCGGTGTGAACGGATTTG  Reverse Primer: TGTAGACCATGTAGTTGAGGTCA |
